# Structure–performance relationships of redox mediators in the lithium–sulfur battery

**DOI:** 10.1039/d6ee01899h

**Published:** 2026-11-09

**Authors:** Ganesh Vailaya, William J. V. Townsend, Alexander J. Kibler, Conrad Holc, Joanne S. Searle, Martin R. Black, Graham N. Newton, Darren A. Walsh, Lee R. Johnson

**Affiliations:** a Nottingham Applied Materials and Interfaces Group, School of Chemistry, University of Nottingham Nottingham NG7 2TU UK graham.newton@nottingham.ac.uk darren.walsh@nottingham.ac.uk lee.johnson@nottingham.ac.uk; b The Faraday Institution, Harwell Campus Didcot OX11 0RA UK; c Advanced Propulsion Lab, Department of Chemical Engineering, University College London London E20 2AE UK

## Abstract

The lithium–sulfur battery is one of the most promising next-generation battery technologies for high-energy applications due to its high gravimetric energy density of 2567 Wh kg^−1^. Despite its advantages, widespread application of the lithium–sulfur battery has been hindered by poor cycle life and incomplete sulfur utilization. Homogeneous redox mediators (RMs) have emerged as effective additives to enhance the capacity of the battery, yet their mechanistic roles and the links between their molecular properties and cell performance remain poorly understood. Here, we describe the structure–performance relationship of a range of redox mediators in these systems, revealing for the first time the sulfur redox reactions driven by the action of the redox mediators. We show that for quinone-based mediators, the potential of the second reduction is crucial in directing the interconversion between sulfur and lithium sulfide. Our results also demonstrate that mediators are not innocent in degradation processes and can induce parasitic reactions at lithium and/or degrade spontaneously. Incorporation of bulky substituents on the mediator structure enhances operational stability by suppressing these degradation routes. We identify 2,6-di-*tert*-butyl-1,4-benzoquinone as an especially promising mediator, delivering the greatest performance gains of the quinone-based mediators we studied, and providing a 16% capacity improvement over the standard after 100 cycles. Our mechanistic insights explain this optimal structure–performance relationship and rationalise why previous use of mediators have resulted in poor coulombic inefficiencies. This work enables predictive design of future RMs with improved stability and electrochemical selectivity, and offers a toolbox for rapid evaluation prior to cycling studies.

## Abstract 2

The lithium–sulfur battery is one of the most promising next-generation battery technologies for high-energy applications due to its high gravimetric energy density of 2567 Wh kg^−1^.

## Graphical abstract

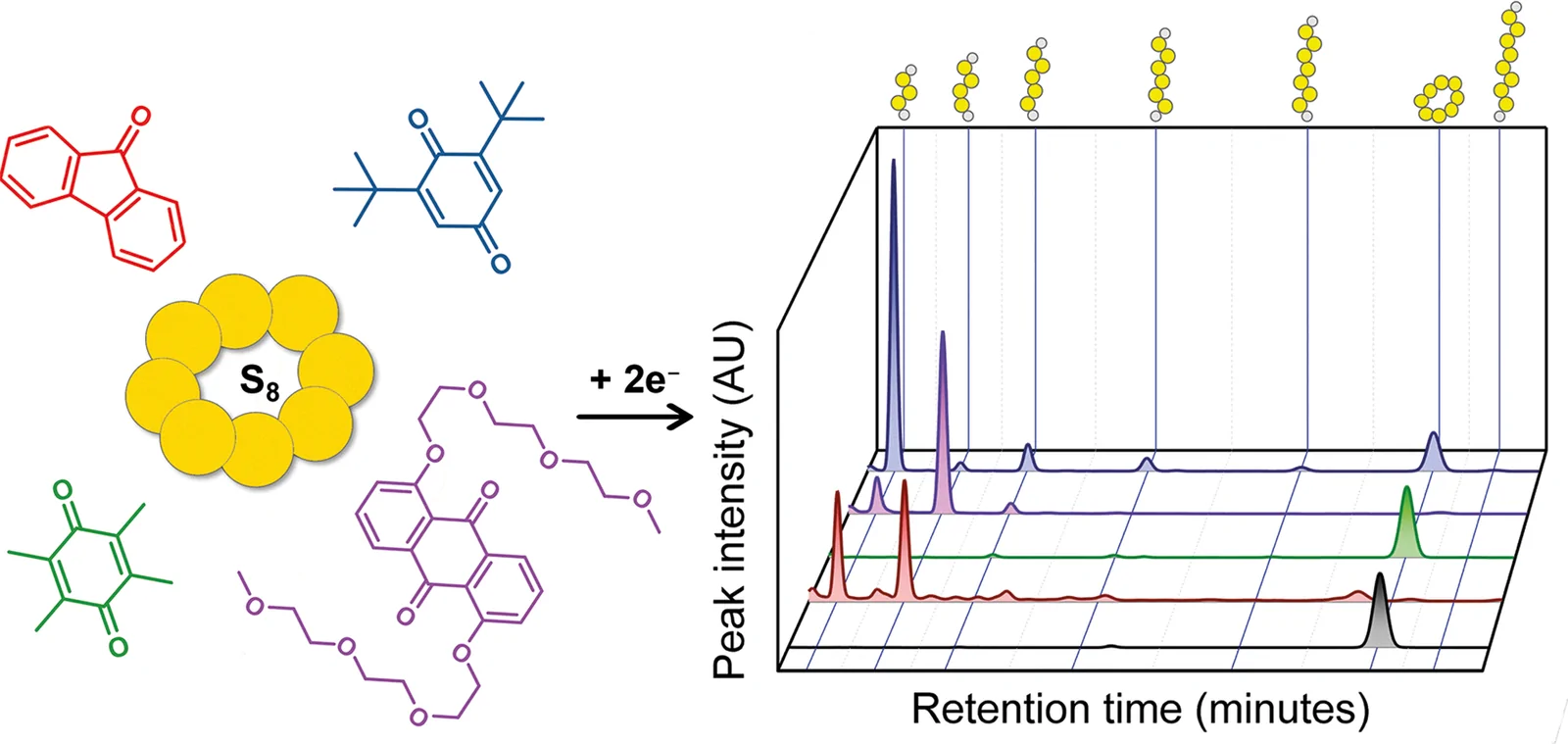

**Broader context**

Commercial lithium-ion batteries are approaching the limits of their practical energy density, falling short of the performance required for energy-intensive applications such as electric aviation, satellites and long-range transport. Lithium–sulfur (Li–S) batteries offer a compelling alternative for next-generation energy storage, with an exceptionally high theoretical specific energy of ∼2500 Wh kg^−1^ and the economic and environmental advantages associated with abundant sulfur. However, key electrochemical and chemical bottlenecks, particularly sluggish conversion between sulfur and lithium sulfide and parasitic reactions at the reactive lithium–metal anode, continue to hinder commercialisation. Redox mediators offer a promising strategy to accelerate sulfur conversion by facilitating rapid electron transfer, but their underlying mechanisms and degradation pathways remain poorly understood. Here, we introduce a comprehensive screening toolkit to evaluate mediator efficacy, establish relationships between mediator redox potential and performance, and identify the structural factors governing mediator stability. By providing actionable design rules for efficient and stable redox mediators, this work advances the rational design of high-energy Li–S batteries and supports their development towards practical high-specific-energy storage.

## Introduction

Lithium–sulfur (Li–S) batteries could offer gravimetric energy densities exceeding 400 Wh kg^−1^ at the system level (originating from a theoretical specific energy density of 2567 Wh kg^−1^) rendering the technology a leading contender for weight-sensitive energy storage applications, such as the electrification of haulage and aerospace.^1–6^ Despite this opportunity, Li–S batteries suffer from rapid capacity fade and low utilization of active material. These complications arise from the low conductivity of the terminal charge/discharge products (S_8_ and Li_2_S) and their redistribution within the 3D electrode structure during cycling, instability of the lithium electrode, and shuttling of highly soluble polysulfide intermediates between the two electrodes.^7,8^ Attempts to address these challenges have focused on the design of the carbon/sulfur composite electrode,^9,10^ modification of the electrolyte formulation,^11,12^ the introduction of heterogeneous catalysts,^13,14^ protection of the lithium electrode,^15^ trapping of the active sulfur within the electrode,^16^ and the use of dissolved, redox-active electrolyte additives known as redox mediators (RMs).^17,18^

RMs can shuttle electrons between the electrode and the active material, which is particularly important when facilitating charge or discharge of an insulating solid. They may also act as catalysts that offer alternative mechanistic pathways beyond simple redox steps.^19^ These types of redox molecule have been widely used in Li–O_2_ batteries,^20,21^ dye sensitised solar cells,^22^ redox flow batteries,^23^ and organic batteries.^24^ When used in Li–S batteries, they can mitigate poor utilization of Li_2_S and S_8_ and inhibit loss of active material by reacting with electrically disconnected sulfur species.^19,25^ Aurbach and co-workers demonstrated that decamethylferrocene could be used as a charging mediator in Li–S batteries. Decamethylferrocene has a highly-positive redox potential (∼2.9 V *vs*. Li^+^|Li) and oxidized Li_2_S, reducing the charging overpotential and improving utilization of Li_2_S.^17^ In recent years, a number of papers have reported the use of quinone-based RMs in Li–S batteries.^18,26–29^ Quinones typically exhibit two redox processes in the operational potential window of the battery, rendering them candidates for mediation of multiple processes during charging and/or discharging. The potential of the RM redox processes can be tuned through simple modification of the quinone backbone.^30^ Peng *et al.* reported a fused anthraquinone structure, 5,7,12,14-pentacenetetrone,^26^ which mediated polysulfide liquid–liquid reactions and the final reduction reaction to Li_2_S. Similarly, Zhu *et al.*^28^ used an anthraquinone in the semiquinone Li^+^ salt (lithium 2-*tert*-butyl-semianthraquinone) as a bidirectional mediator for charging and discharging reactions (2.31 and 2.23 V *vs*. Li^+^|Li). Both additives improved cycling performance under lean electrolyte conditions and at high C rates. Tsao *et al.*^29^ introduced glycol ether pendent groups to anthraquinone to form a highly soluble analogue (1,5-bis(2-(2-(2-methoxyethoxy)ethoxy)ethoxy) anthra-9,10-quinone (AQT)), which operated between ∼2.1 and ∼2.45 V *vs*. Li^+^|Li. Using high additive concentrations of 80 and 160 mM, the team demonstrated Li_2_S cells with good cyclability (>800 mAh g^−1^ S at 100 cycles). Work focusing on the addition of RMs to Li–S cells have frequently used Li_2_S cathodes, as the typical overpotential required on the first charge can be bypassed with the addition of RMs, and these cells could operate with Li-free negative electrodes.^31^

In batteries based on simple reactions with few intermediates, such as the Li–O_2_ battery, detailed understanding of the RM-assisted mechanisms, kinetics and thermodynamics have been reported, allowing for specific understanding of the role of the mediators and thus targeted design of high-performance RMs.^21,32–35^ In contrast, due to the complexities of multi-electron processes that occur during sulfur redox, the link between RM redox potential and specific steps in sulfur/sulfide interconversion remains unclear. Consequently, rather than mediating a single elementary reaction, RM may initiate and participate in a network of coupled chemical and electrochemical sulfur–polysulfide interconversion reactions, with the overall product distribution governed by the thermodynamics and kinetics of the sulfur redox chemistry and the specific RM. There are also unanswered questions regarding the behaviour of RMs at the negative electrode and their compatibility with full Li–S cells. For example, Tsao *et al.* presented an unrationalized drop in the coulombic efficiency (CE) of cells containing AQT when moving from low loading (0.7 mg cm^−2^, average CE >98%) to high loading (6 mg cm^−2^, average CE 92.8%) Li_2_S cathodes.^29^ To realize the full impact of redox mediators in Li–S cells, and establish design principles for high-performance mediators, it is critical to gain a deeper understanding of their role within the cell.

Here we describe the impact of a series of RMs within the Li–S cell and rationalize their influence on performance in terms of their physicochemical properties. We selected a series of RMs with redox potentials spanning the operating voltage range of the Li–S battery (1.8–2.6 V *vs*. Li^+^|Li) and systematically assessed their impact on cell behaviour. Using liquid chromatography-mass spectrometry (LC-MS), we quantified how mediators with distinct redox potentials and archetypal structures drive the interconversion between sulfur and lithium sulfide. The compatibility of RMs with the key cell components, the Li negative electrode and the electrolyte solution, was examined using ultramicroelectrode (UME) voltammetry and nuclear magnetic resonance (NMR) spectroscopy. These complementary analyses reveal that specific degradation and failure pathways can be directly attributed to the presence of certain RMs. We identify the origins of the poor coulombic efficiencies observed in previous studies and show how tailored mediator design can suppress these parasitic processes and deliver substantial improvements in Li–S cell performance. Our studies lead to actionable principles for developing next-generation redox mediators in high-energy Li–S batteries.

## Results and discussion

### Impact of mediator reduction potential on sulfur redox

Fig. 1A shows cyclic voltammograms of RMs in 1.0 M LiTFSI, 0.5 M LiNO_3_ 1 : 1 1,3-dioxolane/1,2-dimethoxyethane, the standard electrolyte solution used in the Li–S battery. RMs were selected such that their redox potentials span the operating potential of the Li–S positive electrode (1.8 V–2.6 V *vs*. Li^+^|Li), enabling us to systematically investigate how mediator redox potential influences sulfur redox chemistry. The quinone scaffold was selected because its two sequential redox processes occur within the operating window of the Li–S positive electrode, making it well suited to mediate multiple sulfur conversion reactions. In addition, varying the degree of substitution around the central quinone ring allowed us to investigate the influence of molecular structure on mediator stability and performance under battery operating conditions. These substituents also influence molecular size and physicochemical properties, such as diffusion, which ideally should be constant, however, these parameters cannot be varied independently from the electronic structure of the quinone core. Specifically, 2,6-di-*tert*-butyl-1,4-benzoquinone (TBBQ) and duroquinone (DQ), which have been used previously in Li–air batteries, and 1,5-bis(2-(2-(2-methoxyethoxy)ethoxy)ethoxy)anthra-9,10-quinone (AQT) which has been reported as an additive in Li–S batteries, were chosen.^29,32,36,37^ All quinone-based RMs displayed two pairs of redox waves. Modifying the pendant group around the central ring gave an expected, but small, change in potential of the first reduction. The largest impact was on the second reduction which counterintuitively shifted to negative potentials when electron-withdrawing pendant groups were added. Based on studies of similar systems, this is expected to lower the electron density on the central ring and oxygens, weakening interaction with the Li^+^ counter ion making the second step more difficult.^30^ Although we note quinone electrochemistry in non-aqueous solvents is complex and multiple factors impact redox potential. We also included 9-fluorenone (9-FO), which was used previously in Li–S cells and undergoes a single one-electron reduction at a more negative redox potential of ∼1.9 V *vs*. Li^+^|Li.^38^ For comparison, Fig. 1A also shows the well-documented voltammetry of S_8_,^39^ which contains two distinct reduction waves at 2.4 V and 2.15 V purported to be due to reduction of S_8_ to S_4_^2−^ and further reduction of S_4_^2−^ to form Li_2_S, respectively. However, we note that these are complex electrochemical processes containing multiple redox reactions.^40,41^ On the return positive scan, Li_2_S and polysulfides were oxidized back to S_8_ at approximately 2.5 V *vs*. Li^+^|Li.

**Fig. 1:**
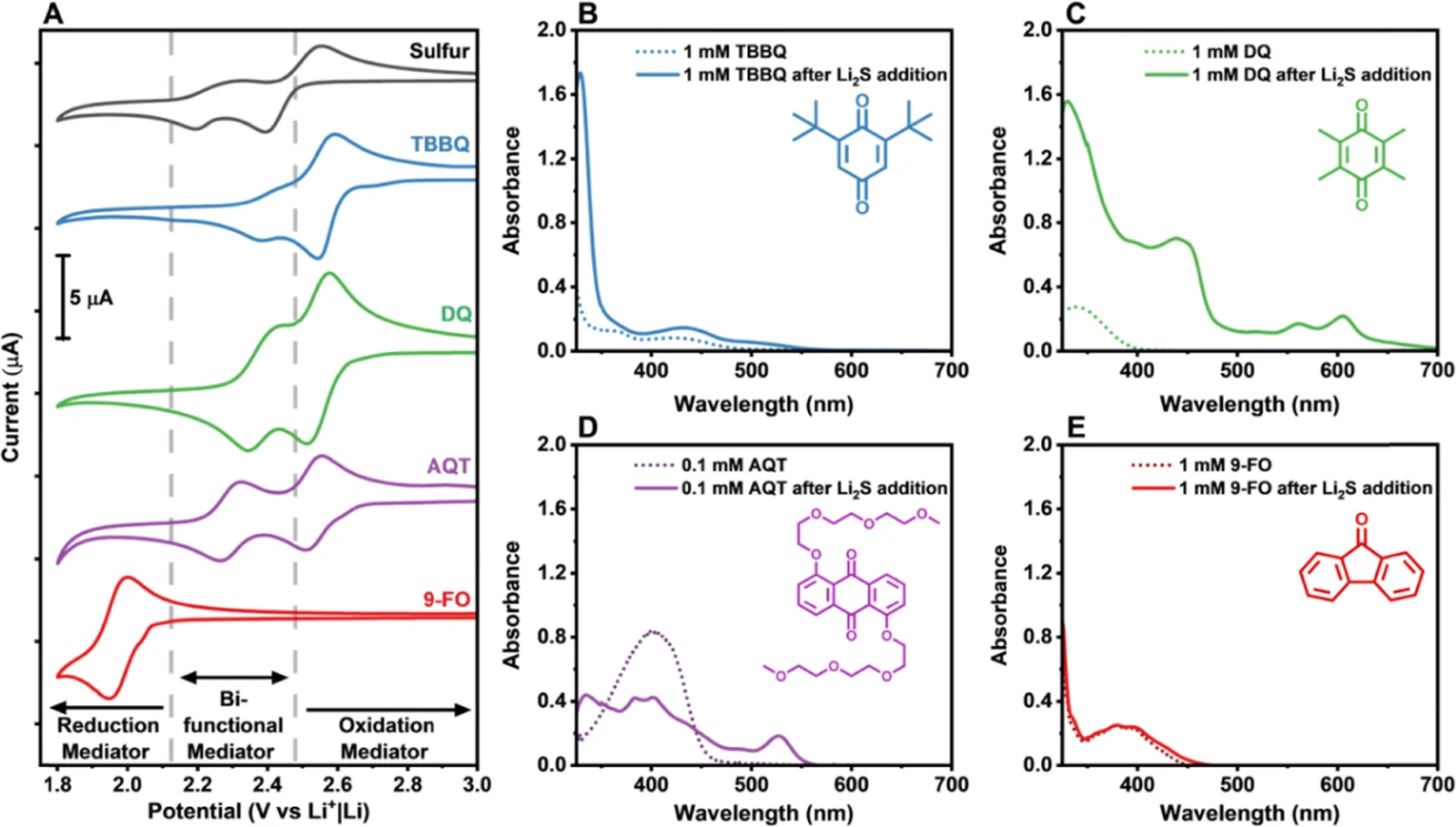
Electrochemical and photochemical characterisation of redox mediators and their impact on sulfur redox reactions. (A) Cyclic voltammograms of 1.0 mM sulfur, 1.0 mM TBBQ, 1.0 mM DQ, 1.0 mM AQT and 1.0 mM 9-FO, recorded using a 3 mm diameter glassy carbon working electrode at a potential scan rate of 10 mV s^−1^, a Pt counter electrode and a Li_1−*x*_FePO_4_ reference electrode. (B)–(E) UV-visible spectra of TBBQ, DQ, AQT and 9-FO solutions in 1.0 M LiTFSI, 0.5 M LiNO_3_ in DOL/DME (1 : 1) before and after addition of Li_2_S. UV-visible spectra of the electrochemically reduced mediators before and after the addition of sulfur can be found in the SI (Fig. S1).

To confirm the ability of the mediators to drive redox reactions within the cell, Li_2_S was added to solutions of the mediators in their oxidized state, and the reactions were monitored using UV-visible spectrophotometry (Fig. 1B–E). Solutions of all mediators showed a broad band between 350–450 nm, corresponding to π → π* transitions. Immediately upon mixing TBBQ, DQ and AQT with Li_2_S, a colour change was observed along with significant changes of the spectral features, indicating rapid oxidation of Li_2_S. This is in contrast to that observed when Li_2_S was added to a solution of 9-FO, when no change was observed indicating that no reaction occurred. These observations are consistent with the reduction potentials of the RMs in comparison to the expected reduction potential of Li_2_S at 2.15 V *vs*. Li^+^|Li (Fig. 1A),^29^ where the RMs with more positive reduction potentials can successfully oxidize Li_2_S while 9-FO, with its lower reduction potential, is unreactive towards Li_2_S. When mixing reduced mediators (formed by bulk electrolysis, see SI for details) with sulfur to assess reduction mediation, significant and rapid changes were observed in the UV-visible spectra, confirming all RMs are able to reduce sulfur (Fig. S1). In all cases where a reaction between the mediator and sulfur species occurred, the spectral changes were effectively instantaneous on the timescale of the experiment, indicating that these chemical reactions are fast relative to the subsequent analytical measurements. Consequently, the differences in product distributions discussed below are attributed to differences in thermodynamic driving force rather than measurable differences in reaction kinetics. Cyclic voltammetry of the mediators in the presence of Li_2_S or sulfur (Fig. S12 and S13) showed no measurable electrocatalytic enhancement during sulfur reduction but a clear increase in anodic current for AQT, DQ and TBBQ mediators in the presence of Li_2_S. The catalytic current becomes most pronounced at potentials where the fully oxidized quinone is expected to predominate, consistent with this species mediating oxidation in the Li–S cell. While UV-visible spectroscopy and cyclic voltammetry confirms that the mediators react with the sulfur species, it is hard to confirm the specific polysulfides formed due to the broad and overlapping absorption bands,^3^ and an alternative technique is required to elucidate which polysulfides are formed upon reaction of RMs and sulfur/lithium sulfide.

### Identification of polysulfides using liquid-chromatography

Polysulfides can be treated with benzyl chloride to yield benzylated polysulfides, limiting exchange of sulfur atoms and trapping the polysulfide distribution for *ex situ* analysis by liquid-chromatography.^42,43^ Adapting this methodology, we analyzed the polysulfides formed when mediators reacted with S_8_ and Li_2_S. Fig. 2A depicts the distribution of polysulfide chains that form when excess AQT^2−^, DQ^2−^, TBBQ^2−^ and 9-FO^−^ (formed by electrochemical reduction) reacted with S_8_. During battery operation the semiquinone (Q˙^−^) will also participate in sulfur conversion but is only indirectly probed here as an intermediate of the RM redox. The assignment of each peak was confirmed by liquid chromatography-mass spectrometry (LC-MS). As expected from the fact that their redox potential(s) are significantly more negative than that of S_8_ reduction (2.48 V *vs*. Li^+^|Li based on Fig. 1A), AQT^2−^ and 9-FO^−^ reduced S_8_ to lower-order polysulfides, as indicated by the near loss of the S_8_ signal. Electrochemically formed TBBQ^2−^ partially converts S_8_ to low-order polysulfides (predominantly S^3−^) while DQ^2−^ showed limited formation of polysulfides.

**Fig. 2:**
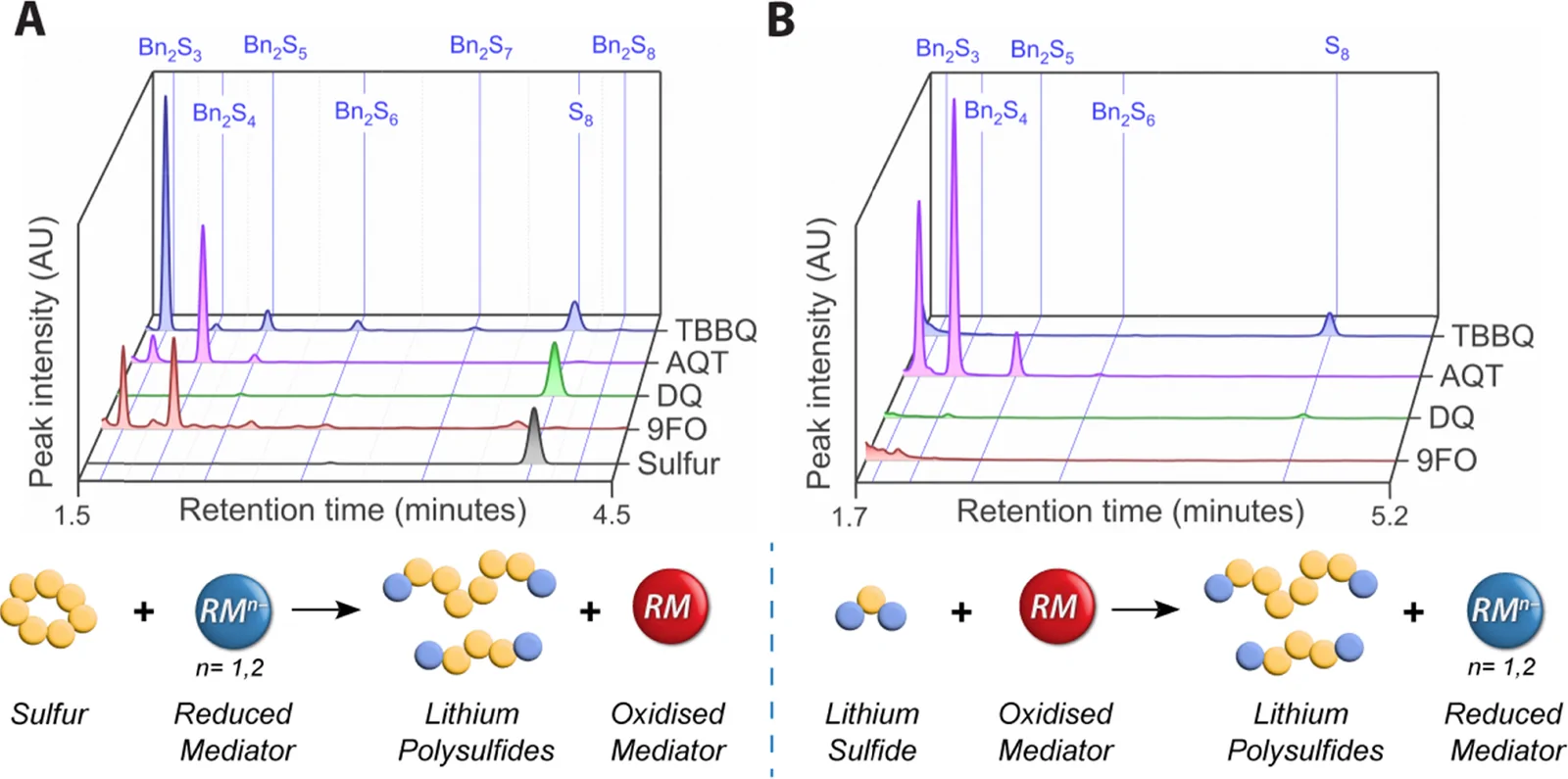
Liquid chromatography analysis of the reactions between redox mediators and S_8_ and Li_2_S using benzyl chloride as a polysulfide-capping agent. Chromatograms of (A) 100 mM excess of electrochemically reduced redox mediators mixed with 12.8 mg (10 mM equivalent) S_8_, and (B) 100 mM excess redox mediators mixed with 2.3 mg (10 mM equivalent) Li_2_S. Samples were prepared for LC-MS by combining 200 µL of the reaction solution with 200 µL BnCl and 1.0 mL methanol.

Fig. 2B shows the extent of Li_2_S oxidation using the mediators in their oxidized state. TBBQ and DQ oxidized Li_2_S to S_8_, and TBBQ was more effective. AQT oxidized Li_2_S to low order polysulfides. Critically, these data highlight the influence of the pendant groups and the associated potential of the quinone's second reduction in governing the conversion of Li_2_S to elemental sulfur; the second reduction of AQT occurs at a relatively negative potential, so is unable to form S_8_, whereas TBBQ which has the most positive second reduction step formed significant amounts of S_8_. Finally, 9-FO, which is reduced at the most negative potential of all mediators was unable to oxidize Li_2_S, as also seen using the UV-visible spectrophotometry (Fig. 1E). To further validate the relationship between quinone redox potentials and sulfur conversion, two additional quinone derivatives (b2G-AQ and AQ) were investigated (Fig. S9 and S14). These compounds exhibited sulfur reduction and Li_2_S oxidation behaviour consistent with their electrochemical properties, further confirming that the potential and accessibility of the second quinone reduction governs the extent of Li_2_S oxidation, while more negative reduction potentials favour sulfur reduction. A full discussion of this analysis can be found in Supplementary Note 1. In summary, these data demonstrate that, rather than acting on a single isolated redox step, RMs participate in a cascade of sulfur redox reactions, facilitating the sequential interconversion of Li_2_S, soluble polysulfides and S_8_. The final sulfur speciation is governed by the thermodynamic driving force provided by the mediator redox potentials.

Redox targeting explains much of the ability of the RMs to react with sulfur species. However, despite its structural similarities to other RMs, DQ is significantly worse than TBBQ at driving sulfur redox reactions, even though UV-visible spectrophotometry indicates that a reaction had occurred. Previous reports suggest that reduced DQ dimerizes, indicating that side reactions may be responsible for its poor sulfur conversion.^44^ To probe the difference between DQ and the other mediators further, we reduced the three quinone-based RMs chemically, re-oxidized them after 4 hours, and then analyzed them by NMR spectroscopy (Fig. S2) and electrospray ionization mass spectrometry (ESI-MS) (Fig. S3), although TBBQ was not suitable for analysis by MS. While all quinones showed some additional peaks, DQ displayed a large number of new resonances between 0.5–2.5 ppm indicating that DQ reacted with itself. This is supported by the presence of the dimer as a sodium adduct at *m*/*z* = 351.1572 in the ESI mass spectrum. In contrast, the mediators with bulky pendent groups on the central ring were more stable and did not show evidence of dimerization. Even though 9-FO does not possess bulky pendant groups, it is not of the benzoquinone family. Therefore, it was not expected to undergo a dimerization reaction.

### Compatibility of redox mediators with metallic Li electrodes

While the catalytic enhancement of RMs in the battery is well documented, possible detrimental effects of their use have received little attention; in particular, shuttling of RMs between the electrodes or its degradation at metallic Li may explain non-ideal coulombic efficiencies observed in some quinone-mediated cells.^28,29^ To explore such effects, the performances of Li–Cu cells containing RMs were compared to those of cells containing no mediators, using the method previously reported by Xiao *et al.* (Fig. 3A and Fig. S4).^45^ While AQT, TBBQ and DQ doped Li–Cu cells showed coulombic efficiencies of ∼99%, consistent with the RM-free cell, the Li–Cu cells doped with 9-FO showed variations in coulombic efficiencies from cycle to cycle, suggesting that the RM interacted with Li and that degradation of the RM may have interfered with stripping/plating events.

**Fig. 3:**
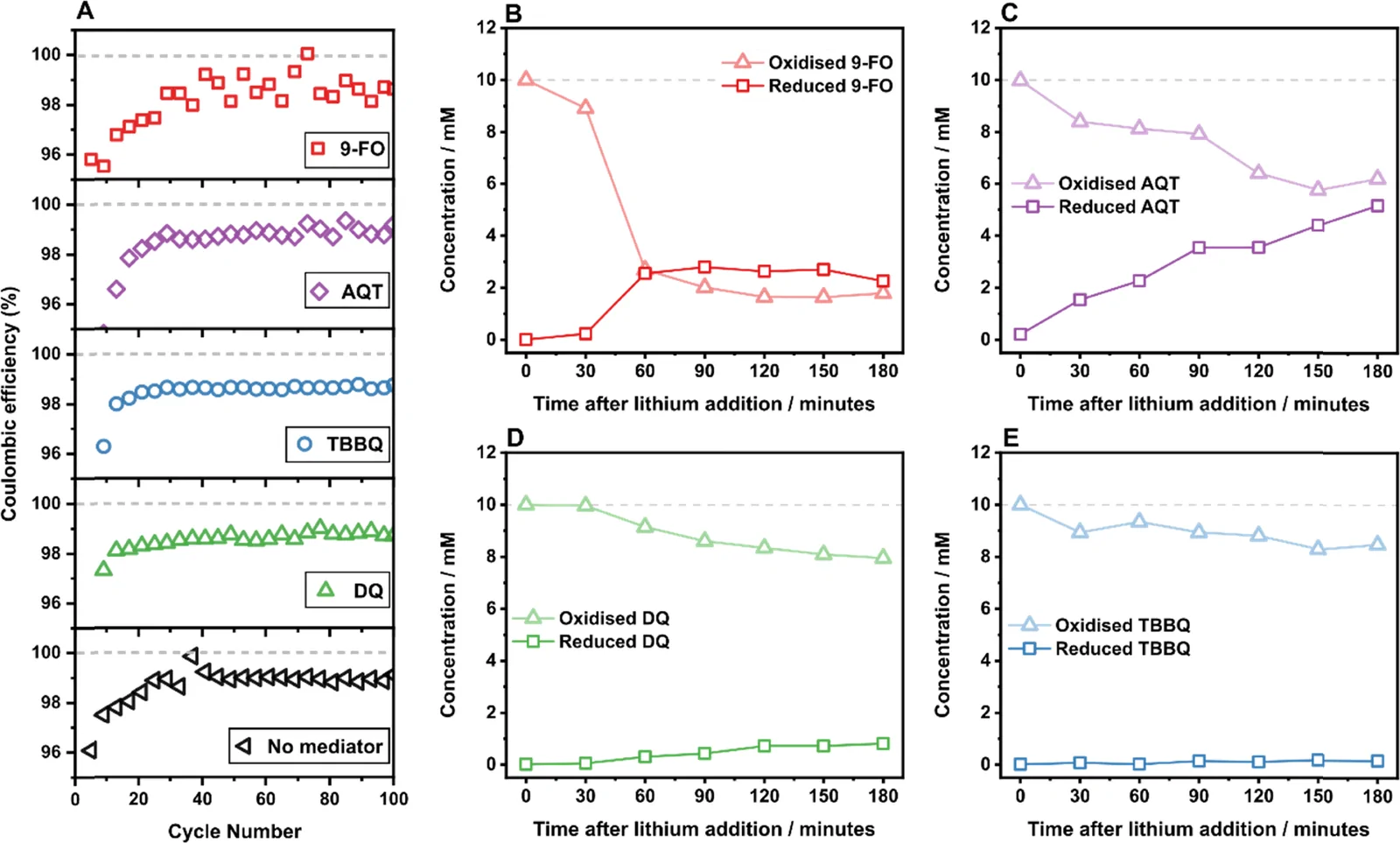
Redox mediator reactions with metallic lithium electrodes. (A) Coulombic efficiencies of Li–Cu cells with 1.0 M LiTFSI, 0.5 M LiNO_3_ in DOL/DME (1 : 1) and cells doped with 50 mM redox mediator, using a current density of 0.13 mA cm^−2^ for 3 cycles and 0.26 mA cm^−2^ for the remainder. Lithium plating (onto copper) was capacity limited and lithium stripping was potential limited.^45^ (B)—(E) Concentration *vs.* time plots monitoring the conversion of neutral mediators to reduced mediators during reaction with Li metal, using 10 mM 9-FO, AQT, DQ and TBBQ. Concentrations were taken from the steady-state currents of voltammograms shown in Fig. S5. The electrolytes consisted of 1.0 M LiTFSI in DOL/DME (1 : 1), the active surface area of lithium was 3.0 cm^2^.

To understand what happened to the mediators when in contact with Li, we carried out ultramicroelectrode (UME) voltammetry of each mediator (10 mM in 1.0 M LiTFSI in DOL/DME, Fig. S5) before and after addition of Li metal to the solutions. The RMs were originally in their oxidized forms, and the concentrations of the oxidized and reduced mediator of interest were extracted from the steady-state currents of these voltammograms over the course of 3 hours (Fig. 3B–E).^46^ The concentration of the oxidized form of 9-FO decreased from 10 mM to 2.7 mM over the first hour. Crucially, this decrease was not matched by a similar increase in the concentration of reduced 9-FO^−^, which would be expected from simple reduction of the RM, and the total concentration was only 5.2 mM after one hour. This confirms that 9-FO reacted irreversibly with Li, as was also indicated during analysis of the 9-FO-containing Li–Cu cells (Fig. 3A).

After addition of lithium to the solution containing AQT, a steady drop in the concentration of the oxidized species occurred over three hours as AQT was reduced at the Li surface (Fig. 3C). In contrast to that observed for 9-FO, the concentration of the reduced AQT increased as that of the oxidized form decreased, and the total concentration of AQT stayed at ∼10 mM, showing that the RM did not degrade on contact with Li. The reactions between Li and DQ or TBBQ (Fig. 3D and 3E, respectively) were slower than that between AQT and Li. A small amount of each appeared to have been lost over the three-hour period but the loss was much less than that of 9-FO, highlighting the importance of the stability provided by the two oxygen centres of the quinones, over that of the single oxygen site in 9-FO. To understand whether the faster reduction of AQT was specific to the anthraquinone structure or due to the addition of the polyether pendent groups, the measurement was repeated using anthraquinone as a control species (Fig. S6). The reaction between anthraquinone and Li was much slower than between AQT and Li, demonstrating that the pendent polyether groups contributed to the faster reduction. To further probe this effect, the experiment was repeated using b2G-AQ, an anthraquinone derivative bearing shorter polyether substituents (Fig. S10). The reduction of b2G-AQ by Li was slower than with AQT, consistent with the polyether substituents contributing to the enhanced reactivity with Li metal. Due to the complexity of the lithium solid/electrolyte interphase and its interaction with the RM, the reason for this difference in rate is unclear, but we suggest this may be due to differences in dissolution rate of AQT and the other quinones stemming from its enhanced interaction with the electrolyte solution.

Shuttling of RMs between electrodes is a known cause of capacity fade in batteries.^47^ To study this phenomenon in our systems, we constructed Li–carbon cells (without any sulfur but containing RMs in the electrolytes) and tested the effects of applying oxidizing potentials to the RMs at the carbon electrode, which when coupled to reduction at the lithium negative electrode, the amount of shuttling between the two by the mediators can be measured (Fig. S7). The 9-FO- and DQ-containing Li–carbon cells showed large decreases in capacity, which is consistent with the instability of each RM in the presence of Li described above. In contrast, TBBQ- and AQT-containing cells were relatively stable. When using current densities typical for Li_2_S cells, the time taken for each cycle decreased drastically from >5 hours to ∼10 minutes, due to the very small capacity of the mediators. Under a short measurement timeframe, the impact of RM shuttling on capacity is likely to be reduced. Therefore, AQT- and TBBQ-containing cells were cycled at decreasing C rates, to more closely reproduce the charge and discharge duration in Li–S cells (Fig. 4B). Upon decreasing the C rate, the TBBQ-containing cell had a stable capacity and CE, while the CE and capacity of the AQT-containing cell decreased and increased, respectively, with each decrease in C rate. These effects are consistent with shuttling of AQT between the electrodes and its rapid reaction at metallic lithium seen in Fig. 3C. During charging, oxidized AQT travels to the lithium electrode and is reduced, after which it travels backs to the positive electrode, resulting in an overcharge and decreasing CE. Over many cycles, this can contribute to slippage, capacity fade and early cell death.

**Fig. 4:**
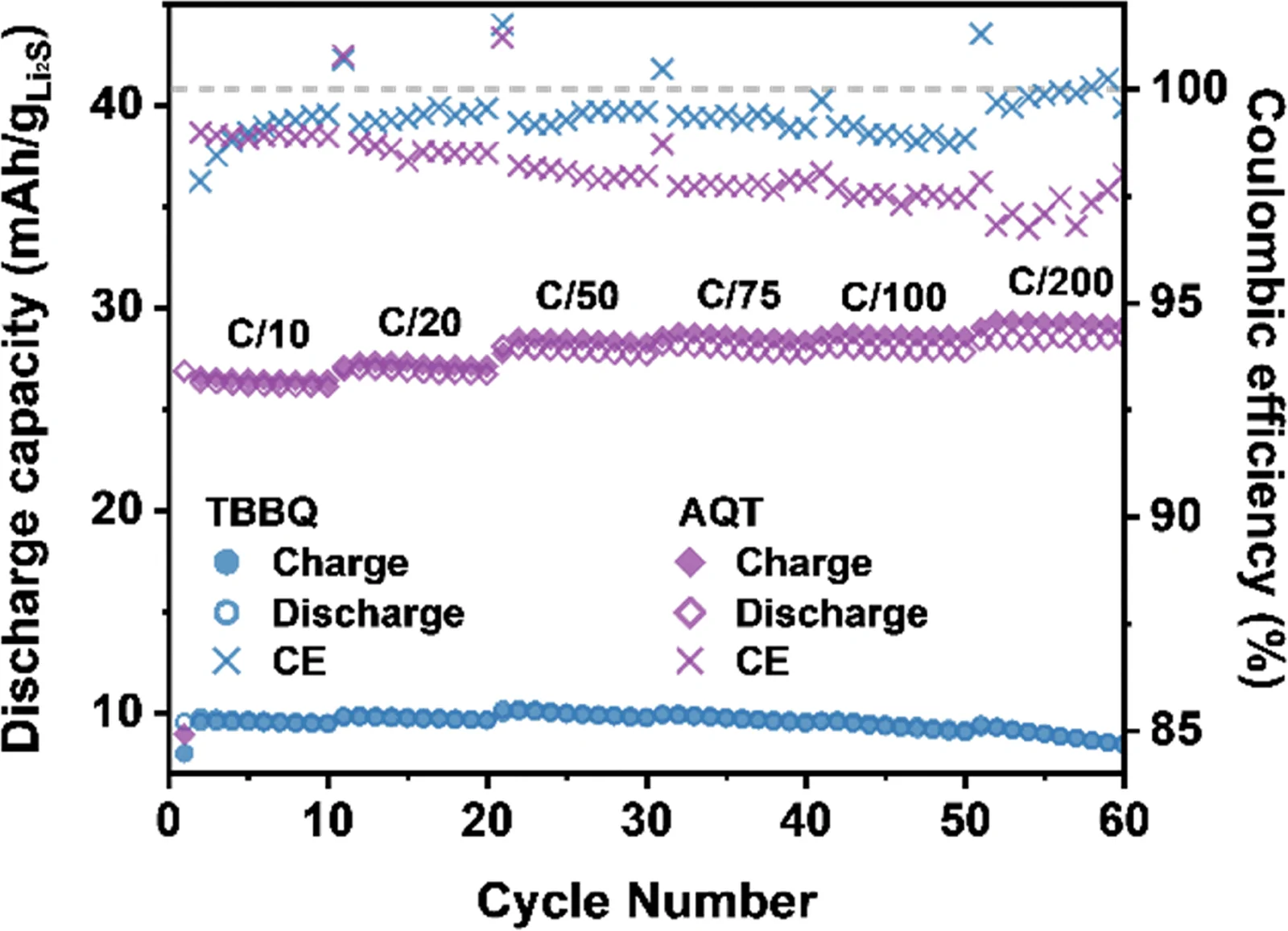
Mediator-doped Li–C cells showing that AQT undergoes significant shuttling. Discharge capacities and coulombic efficiencies of Li–carbon cells, doped with 50 mM of TBBQ and AQT using varied C-rates. In all experiments, the electrolyte solution was 1.0 M LiTFSI, 0.5 M LiNO_3_ in DOL/DME (1 : 1). The C rates for all Li–carbon cells are based on the capacity obtained from a Li_2_S cell with a loading of 2.5 mg.

### Impact of redox mediators on Li–S full cells

Having explored compatibility of RMs with metallic Li and their shuttling behaviour, the RMs were incorporated into Li–Li_2_S cells, an approach taken by others when studying the impact of RMs on cell performance.^29,48,49^ Cells were cycled at C/20 for three cycles and then C/10 unless otherwise stated to minimise transport limitations and isolate the influence of redox mediator chemistry on sulfur conversion and cell stability. Unmediated Li–Li_2_S cells required a large overpotential during initial charging to activate the insulating Li_2_S microparticles (Fig. 5A). The cells containing DQ, TBBQ and AQT, all three of which were able to oxidise Li_2_S, showed lower initial charging overpotentials, due to the RM assisted oxidation of Li_2_S. In contrast, initial charging of cells containing 9-FO required an overpotential higher than even the voltage limit of a typical sulfur cell, which we ascribe to its instability at lithium metal (Fig. S8). When using typical voltage limits, Li_2_S cells with 9-FO routinely failed and 9-FO-mediated cells required a higher voltage window for the first charging process. Despite this, all RM assisted cells showed an increase in capacity compared to unmediated Li_2_S cells in early cycles (Fig. 5B and C). While 9-FO was unable to assist with Li_2_S charging, once the Li_2_S was activated, the 9-FO may act as a reduction mediator for the reduction of sulfur, resulting in the observed capacity increase. Despite this initial capacity increase, 9-FO, DQ and AQT cells demonstrated capacity fade, with the latter also exhibiting poor CE (Fig. 5B–D). Each of these capacity fades can be rationalized using the experimental insights gained so far. In the case of 9-FO and DQ, this capacity fade is due mediator degradation on contact with Li or due to an intrinsic instability. For AQT, the poor coulombic efficiency and drop-off in activity can be attributed to the shuttling of the mediator, as demonstrated in the Li–C cells. Conversely, TBBQ was able to increase capacity of the Li_2_S cells in comparison to standard Li_2_S cells, providing a 16% capacity improvement over the standard after 100 cycles, which is consistent with its ability to drive sulfur redox reactions, stability in the cell conditions, and lack of significant redox shuttling.

**Fig. 5:**
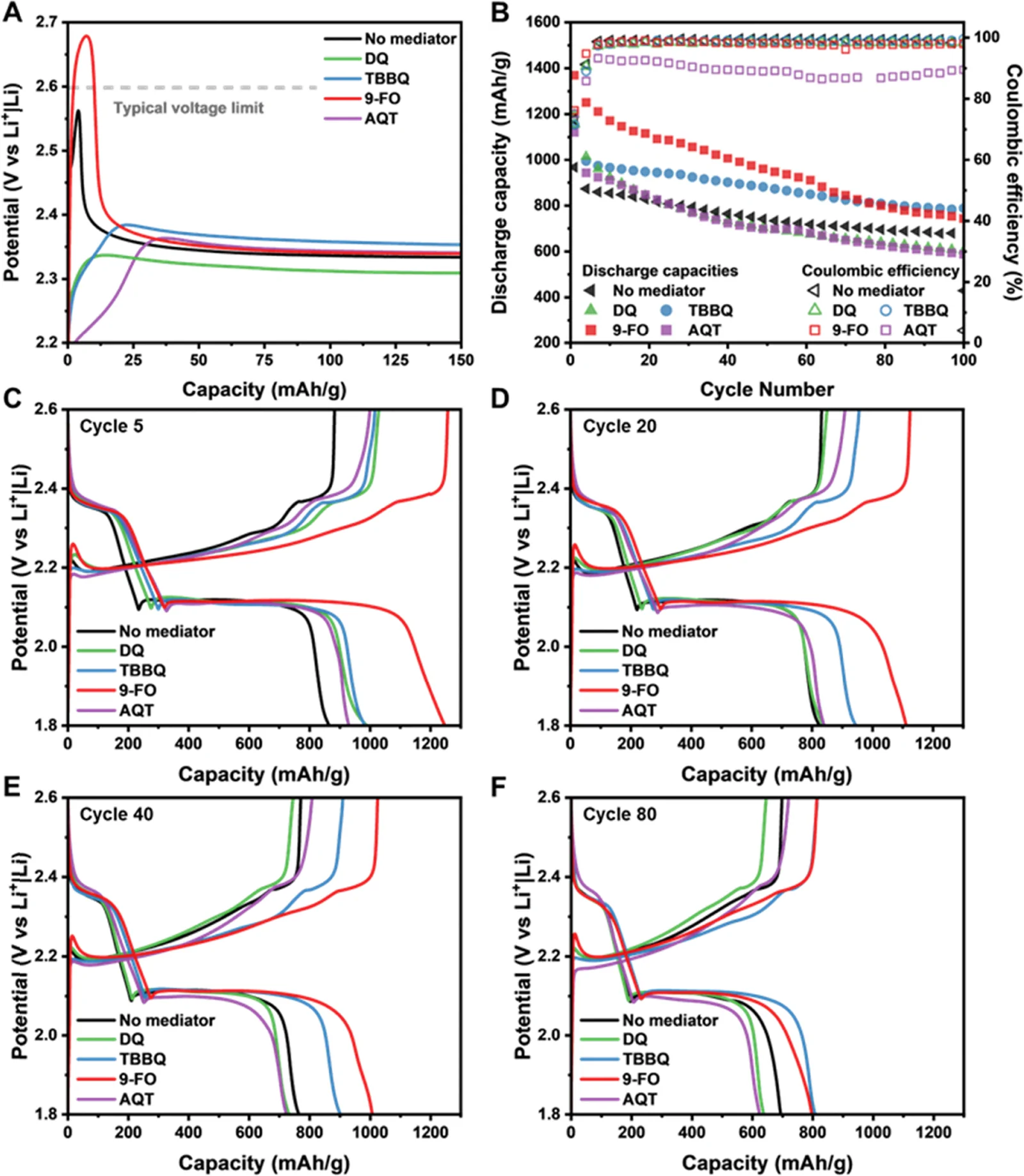
Cell performance of Li–Li_2_S cells with redox mediators, showing TBBQ provides a sustained performance enhancement. (A) First charge of Li–Li_2_S cells with and without redox mediators. (B) Discharge capacities and coulombic efficiencies of Li–Li_2_S cells with and without redox mediators. (C) Cycle 5, (D) 20, (E) 40 and (F) 80 for the cells shown in (B). All mass loadings were approximately 2.5 mg μ-Li_2_S and the electrolyte-to-sulfur ratio was 20 µL mg^−1^. The electrolyte comprised 1.0 M LiTFSI, 0.5 M LiNO_3_ in DOL/DME (1 : 1), with addition of 50 mM of either DQ, TBBQ. 9-FO or AQT. All cells were cycled at 0.05C for 3 cycles and 0.1C for the remaining cycles with voltage limits of 1.8 and 2.6 V *vs.* Li^+^|Li, apart from 9-FO-containing cells, which had an upper voltage limit of 2.7 V *vs*. Li^+^|Li during the first cycle. Statistical analysis of the discharge capacities is provided in Fig. S11.

The oxidation state of the quinone mediators is also expected to evolve throughout the charge and discharge process as the cell potential traverses their two redox couples (Fig. 1A). During discharge, the mediators are initially present predominantly in their oxidized quinone form (Q), with reduction to the semiquinone radical anion (Q˙^−^) and subsequently the dianion (Q^2−^) becoming progressively more favourable as the cell potential decreases. Consequently, the mediators are expected to have relatively little influence during the initial reduction of S_8_ to higher-order polysulfides but to become increasingly active during the lower discharge plateau, where conversion of soluble polysulfides to insulating Li_2_S_2_/Li_2_S is kinetically sluggish, which can be seen in the extended second plateau when mediators are present. During charging, the reverse sequence is expected. At the lower charging plateau (~2.2–2.3 V *vs*. Li^+^|Li), the mediators are anticipated to exist predominantly in their reduced forms (Q^2−^ and Q˙^−^), enabling chemical oxidation of Li_2_S and lower-order polysulfides. As the charging potential increases, the equilibrium shifts towards the oxidized quinone, increasing the driving force for oxidation of higher-order polysulfides ultimately to S_8_, which again can be seen as an increased capacity for the second plateau during charging with mediators. The extent to which this occurs is governed by the second quinone reduction potential, consistent with the LC-MS results showing that TBBQ, which possesses the most positive second reduction, promotes extensive conversion to S_8_, whereas AQT, with a significantly more negative second reduction, predominantly yields lower-order polysulfides.

## Conclusions

Our results highlight the critical influence of redox mediator molecular structure on performance and stability in Li–S batteries. The strong correlation between analytical characterization and cell performance demonstrates that predictive, structure-guided design of redox mediators for Li–S systems is now achievable. Our systematic comparison shows that mediator performance is governed by three interrelated factors: (i) redox potentials that are appropriately matched to the sulfur conversion reactions of interest, (ii) molecular stability towards degradation under battery operating conditions, and (iii) compatibility with the lithium negative electrode, including suppression of mediator shuttling. Although these properties cannot be varied independently through molecular design, the combination of electrochemical, spectroscopic and cell-based measurements presented here enables their individual contributions to be identified. Among the mediators studied, 2,6-di-*tert*-butyl-1,4-benzoquinone (TBBQ) delivered the most pronounced performance improvements, validating the mechanistic rationale established here. Its superior performance arises from the combination of favourable redox properties, which enable efficient sulfur–lithium sulfide interconversion, together with resistance to degradation and minimal mediator shuttling under battery operating conditions.

This work provides a clear framework for the future design of stable and selective redox mediators. Effective mediators must simultaneously possess redox potentials matched to the desired sulfur conversion reactions, sufficient structural stability to suppress parasitic degradation, and minimal reactivity with the lithium negative electrode. Future candidates should focus on the quinone scaffold due to its inherent stability and redox potential, which lies within the optimal operating range for Li–S batteries. Modification of the quinone core with pendant substituents must balance the competing effects of charge distribution and Li^+^ coordination, ensuring that both redox events occur at similar potentials while kinetically inhibiting dimerization and other radical-driven degradation pathways. In practice, the incorporation of bulky, electron-donating *tert*-butyl groups appears to offer the twin benefits of optimized redox potentials and suppression of parasitic reactions within the family of mediators investigated here. While increasing solubility, for example with polyether chains, will be important, such modifications can have additional consequences such as enhanced reactivity with Li metal and further study is required. The modest capacity improvements observed in this work highlight that a single redox mediator is unlikely to optimise every stage of the complex sulfur conversion pathway. An attractive future strategy is the development of complementary mediator systems with redox potentials strategically distributed across the Li–S operating window, allowing different mediators to selectively accelerate individual sulfur–polysulfide conversion reactions. Finally, the analytical approach demonstrated here can serve as a practical toolbox for evaluating redox mediators prior to cycling and without relying solely on post-mortem electrode analysis, providing a robust down-selection strategy applicable across a range of electrochemical energy systems.

## Author contributions

All authors contributed to the conception and design of the study. G. V., W. J. V. T., A. J. K., C. H., J. S. S. and M. R. B. performed the experiments. All authors contributed to manuscript writing. D. A. W., G. N. N. and L. R. J. supervised the project.

## Conflicts of interest

The authors declare no competing interests.

## Supplementary Material

EE-OLF-D6EE01899H-s001

## Data Availability

The data that support the findings of this study are available from the corresponding author, L. R. J, upon reasonable request. Supplementary information (SI): document S1. Supplemental experimental procedures, Fig. S1–S14. See DOI: https://doi.org/10.1039/d6ee01899h.
