# Supplementary material for: Structure–performance relationships of redox mediators in the lithium–sulfur battery
Source: Energy Environ Sci. 2026 Nov 9. Online ahead of print. doi: 10.1039/d6ee01899h (PMC13746294; doi:10.1039/d6ee01899h)
Supplement: Supplementary file 1 [file EE-OLF-D6EE01899H-s001.pdf]

Supplementary Information (SI) for Energy & Environmental Science.
This journal is © The Royal Society of Chemistry

Structure-Performance Relationships of Redox Mediators in the Lithium-Sulfur Battery

SUPPLEMENTAL INFORMATION

Ganesh Vailaya,[1] William J. V. Townsend,[1,2] Alexander J. Kibler,[2,3] Conrad Holc,[1,2] Joanne S. Searle,[1] Martin R. Black,[1,2] Graham N. Newton,[1,2]* Darren A. Walsh,[1,2]** Lee R. Johnson[1,2,4]***

[1] Nottingham Applied Materials and Interfaces Group, School of Chemistry, University of Nottingham, Nottingham, NG7 2TU, UK

[2] The Faraday Institution, Harwell Campus, Didcot, OX11 0RA, UK

[3] Advanced Propulsion Lab, Department of Chemical Engineering, University College London, London, E20 2AE, UK

[4] Lead Contact

*Correspondence: graham.newton@nottingham.ac.uk

**Correspondence: darren.walsh@nottingham.ac.uk

*** Correspondence: lee.johnson@nottingham.ac.uk

EXPERIMENTAL PROCEDURES

Materials preparation

LiTFSI (99.95% trace metals basis) was purchased from Sigma Aldrich and was dried at 120 °C under vacuum for 72 hours. $LiNO_3$ (99.99% trace metals basis) was purchased from Sigma Aldrich and dried at 70 °C under vacuum for 72 hours. 1,2-Dimethoxyethane (99.5%, anhydrous, inhibitor-free) and 1,3-dioxolane (99.8%, anhydrous, ~75 ppm BHT as inhibitor) were purchased from Sigma Aldrich, dried under 4 Å molecular sieves and stored under N_2. 2,6-*Di*-*tert*-butyl-1,4-benzoquinone, duroquinone and 9-fluorenone were purchased from Sigma Aldrich and recrystallised in ethanol. 1,5-Bis(2-(2-(2-methoxyethoxy)ethoxy)ethoxy) anthra-9,10-quinone was synthesised following procedure in literature.[1] Commercial Li_2S

(99.98% trace metal basis) was purchased from Sigma Aldrich and was used as is. Commercial sulfur (99.998% trace metal basis) was purchased from Sigma Aldrich and was used as is. Lithium ribbon was purchased from Sigma Aldrich (99.9% trace metal basis).

μ-Li_2S synthesis

μ-Li_2S was synthesised following a method described in the literature.[1] Sulfur (320 mg, 1.25 mmol) was dissolved in toluene (17.5 mL) at 40 °C. The sulfur-toluene mixture was then added to a solution of 1.0 M lithium triethylborohydride in tetrahydrofuran (21 mL). The solution was stirred for 2 minutes at room temperature, and then for 7 minutes at 90 °C, producing a cream precipitate. The resulting crude product was collected and washed twice with tetrahydrofuran and hexane. The product was dried at 400 °C for 30 minutes in a N_2 glovebox, leaving a grey solid.

Preparation of reduced quinones

To a solution of the quinone (0.250 mmol, 1.0 eq.) in DOL (2.5 mL) and DME (2.5 mL) was added $NaBH_4$ (9.5 mg, 0.250 mmol, 1.0 eq.) and the mixture stirred. After 4 hours, methanol (1.2 mL) was added to the mixture. The product was dried at 40 °C under vacuum for 48 hours to afford an oil.

Preparation of lithium iron (II) phosphate reference electrode

1 g of carbon black (Alfa Aesar) was mixed with 8 g of $LiFePO_4$ (MTI), 10 g of 10% wt. of polytetrafluoroethylene (PTFE) (Sigma Aldrich), and a small amount of isopropanol to form a putty. The putty was smeared onto a folded steel mesh to create the freestanding electrode. The electrode was then immersed in a solution comprising of 3.6 mL of hydrogen peroxide, 2.5 mL of acetic acid and 250 mL of ultrapure water for 15 minutes to pre-charge the electrode. The electrode was washed with a 1:1 solution of ultrapure water and isopropanol and was left to dry in a vacuum oven for 24 hours at 120 °C. The electrode was then placed behind a frit, assembled in a glovebox and left to soak in a solution of 1.0 M tetrabutylammonium perchlorate and 0.1 M lithium perchlorate in dimethyl sulfoxide.

Cyclic voltammetry

A three-electrode system was used for all cyclic voltammetry measurements. The working electrode was glassy carbon, the counter electrode was platinum wire, and the reference electrode was lithium iron phosphate (LFP) behind a frit. All cyclic voltammetry measurements were conducted in a N_2 glovebox (O_2 < 1 ppm, H_2O < 1 ppm), using an SP-300 Biologic potentiostat. The software used to analyse the data was EC-Lab. The electrolyte composition consisted of 1.0 M LiTFSI, 0.5 M $LiNO_3$ in DOL/DME (1:1 v/v). All cyclic voltammograms are reported vs $Li^+|Li$. The reference potential was converted from LFP to $Li^+|Li$. An iR correction was applied to all cyclic voltammograms.

Bulk electrolysis

Bulk electrolysis was conducted using an SP-300 Biologic potentiostat under a N_2-atmosphere glovebox. The analyte of varying concentrations was dissolved in 1.0 M LiTFSI in DOL/DME (1:1 v/v). A three-neck flask was employed to contain the platinum mesh working electrode and LFP counter and reference electrodes. All analytes were reduced to their lowest oxidation state, to approx. >90%.

Liquid chromatography

A C18-reversed phase silica gel column was used, coupled with a 90% methanol, 9.9% water and 0.1% formic acid mobile phase under isocratic conditions at 45 °C. 5 µL of sample was injected per run and the flow rate was 1.5 mL min^{-1} with a total run time of 12 mins. For sample preparation, 10 mM equivalent of Li_2S or S_8 was reacted with excess 100 mM mediator/reduced mediator for 12 hours under an inert atmosphere. 200 µL of the reacted mixture was combined with 200 µL of benzyl chloride and 1 mL of HPLC grade methanol for LC measurements.
The wavelength used for detecting polysulfides was 254 nm. Benzyl polysulfides were assigned using LC-MS in positive mode by diluting the LC mixtures further with HPLC grade methanol by a factor of 20.

Cathode fabrication

Li_2S cathodes were fabricated by grinding 70 mg of µ-Li_2S and 20 mg of Super-P carbon black for 30 minutes, forming a grey mixture. Separately, 10 mg polyvinylidene fluoride (PVDF) was dissolved in 1.2 mL of dimethylformamide (DMF). This solution was added to the Li_2S/CB

mixture, and stirred overnight, forming a black slurry. 60 μL of the slurry was dropcast onto 12 mm diameter GDL disks and left to dry at room temperature overnight.

Galvanostatic cycling

Galvanostatic cycling with potential limitation was used to discharge and charge Swagelok cells and coin cells. A conditioning step was applied to all Li-S cells, at 0.05 C for 3 cycles. The remaining cycles were set at various C-rates, usually 0.1 C. A Biologic BCS-805/810 series cycler or an Ivium IviCycle was used to cycle the cells.

Coin cell assembly

Li-Cu coin cells (CR2032) were assembled using 12 mm diameter copper disks as the positive electrode, 14 mm diameter lithium metal disks (Sigma-Aldrich) as the negative electrode, separated by a 15 mm diameter Celgard® 2400 separator. 50 μL of electrolyte, consisting of 1.0 M LiTFSI, 0.5 M $LiNO_3$ in DOL/DME (1:1 v/v) and 50 mM of any relevant mediator, was added. All cell assembly was conducted in an Ar glovebox (O_2 <1 ppm, H_2O <1 ppm). Li-GDL coin cells (CR2032) were assembled using 12 mm diameter GDL disks, (Freudenberg H23, Quintech) as the positive electrode, 14 mm diameter lithium disks as the negative electrode and a 15 mm diameter Celgard® 2400 disk as the separator. The electrolyte consisted of 1.0 M LiTFSI, 0.5 M $LiNO_3$ in DOL/DME (1:1 v/v), and was added at an electrolyte to Li_2S ratio of 20 μL mg^{-1}. All cell assembly was conducted in an Ar glovebox (O_2 <1 ppm, H_2O <1 ppm). Li-Li_2S coin cells (CR2032) were assembled using 12 mm diameter Li_2S on GDL disks as the positive electrode, 14 mm diameter lithium metal disks as the negative electrode, separated by a 15 mm diameter Celgard® 2400 separator. An electrolyte to Li_2S ratio of 20 μL mg^{-1} was utilized for all cells. An average mass loading of 2.5 mg was used for these cells. All capacities are normalised to the theoretical sulfur capacity of 1672 mAh g^{-1}. All cell assembly was conducted in an Ar glovebox (O_2 <1 ppm, H_2O <1 ppm).

Supplemental Figures

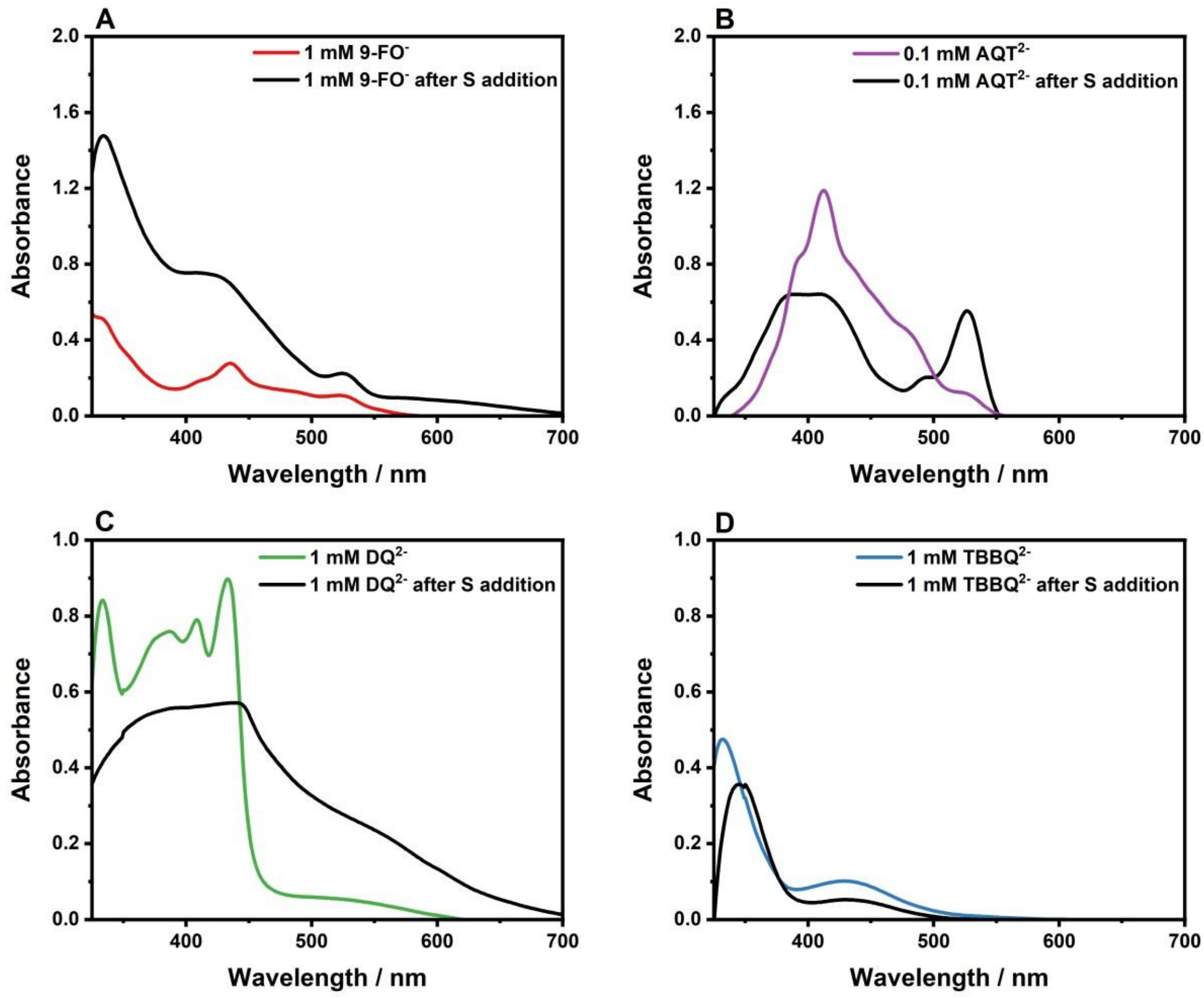

Figure S1. UV-visible spectra of electrochemically reduced RM before and after the addition of sulfur. (A) 1 mM 9-FO$^-$, (B) 0.1 mM AQT^{2-}, (C) 1 mM DQ^{2-} and (D) 1 mM TBBQ^{2-} in 1.0 M LiTFSI, 0.5 M $LiNO_3$ in DOL/DME (1:1 v/v) before and after addition of excess sulfur.

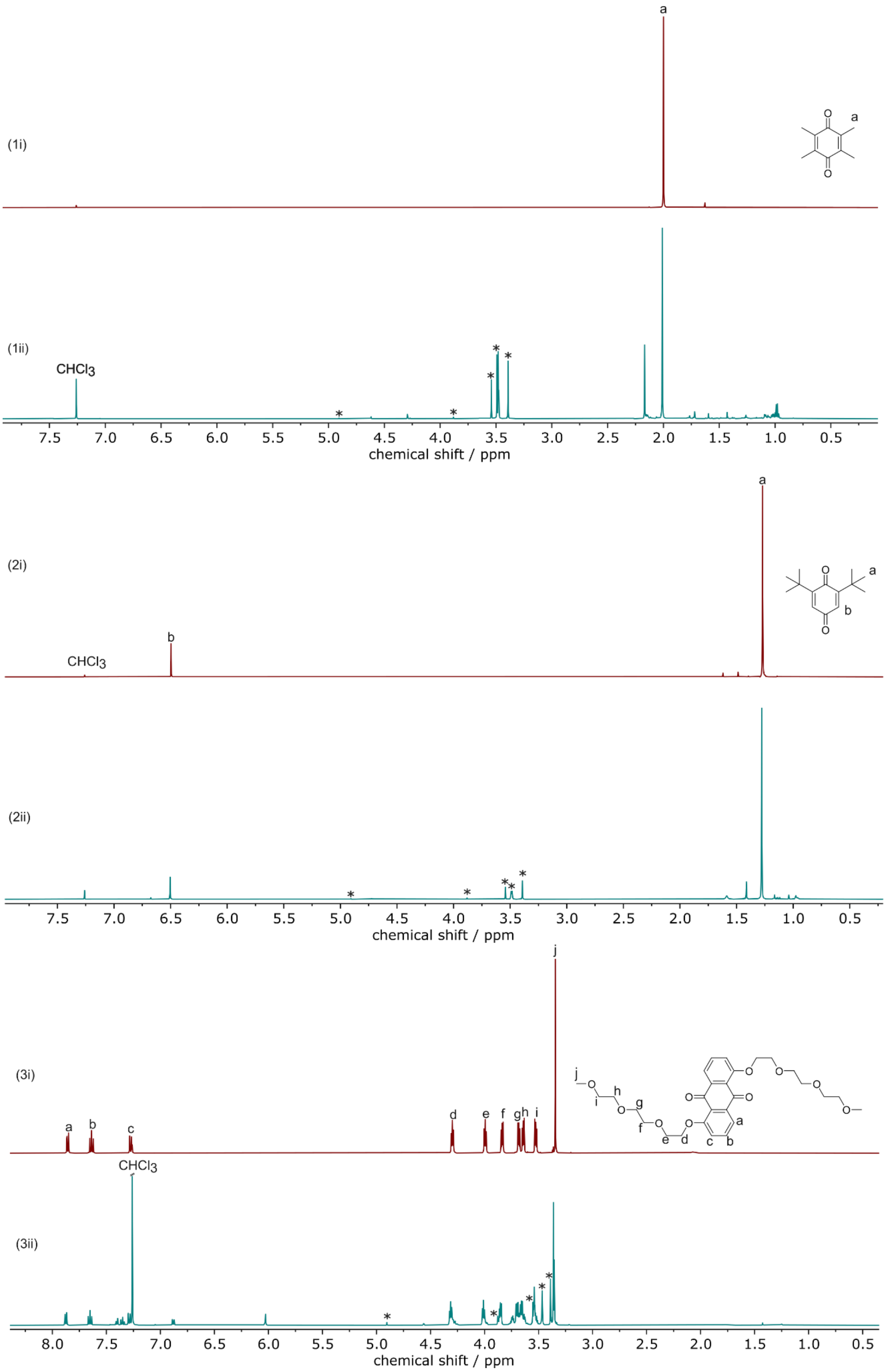

Figure S2. NMR spectroscopy study of reduced mediator stability. DQ before reduction (1i) and after reduction (1ii), TBBQ before reduction (2i) and after reduction (2ii), AQT before reduction (3i) and after reduction (3ii). Each redox mediator was allowed to react in its reduced form for 4 hours before reoxidation and analysed by NMR spectroscopy. Residual solvent signals highlighted by *.

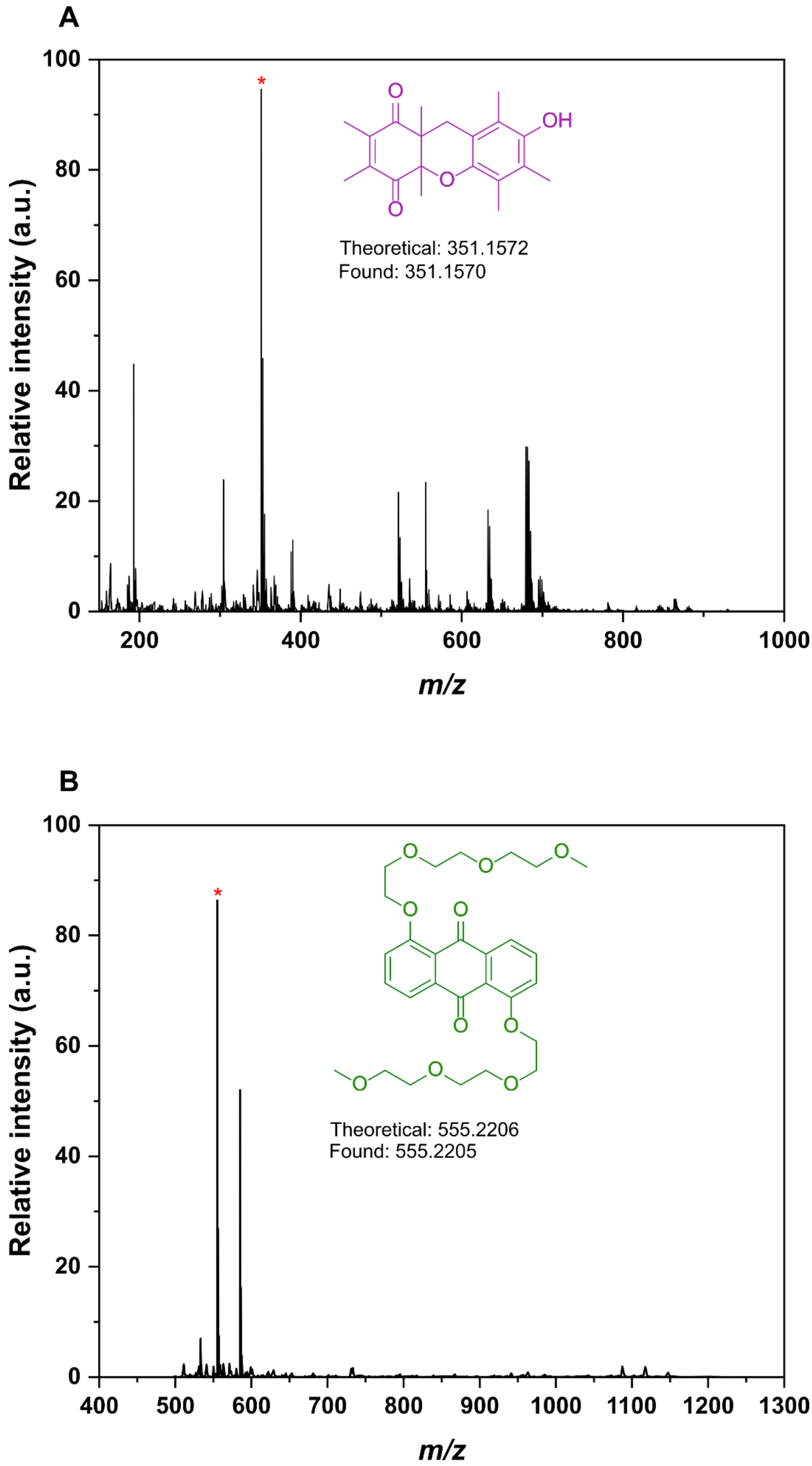

Figure S3. MS analysis of reduced quinones showing dimerization of DQ as a sodium adduct and no dimer formation for AQT. MS of (A) DQ and (B) AQT after reduction using $NaBH_4$. TBBQ gave weak signals making it unsuitable for analysis by MS.

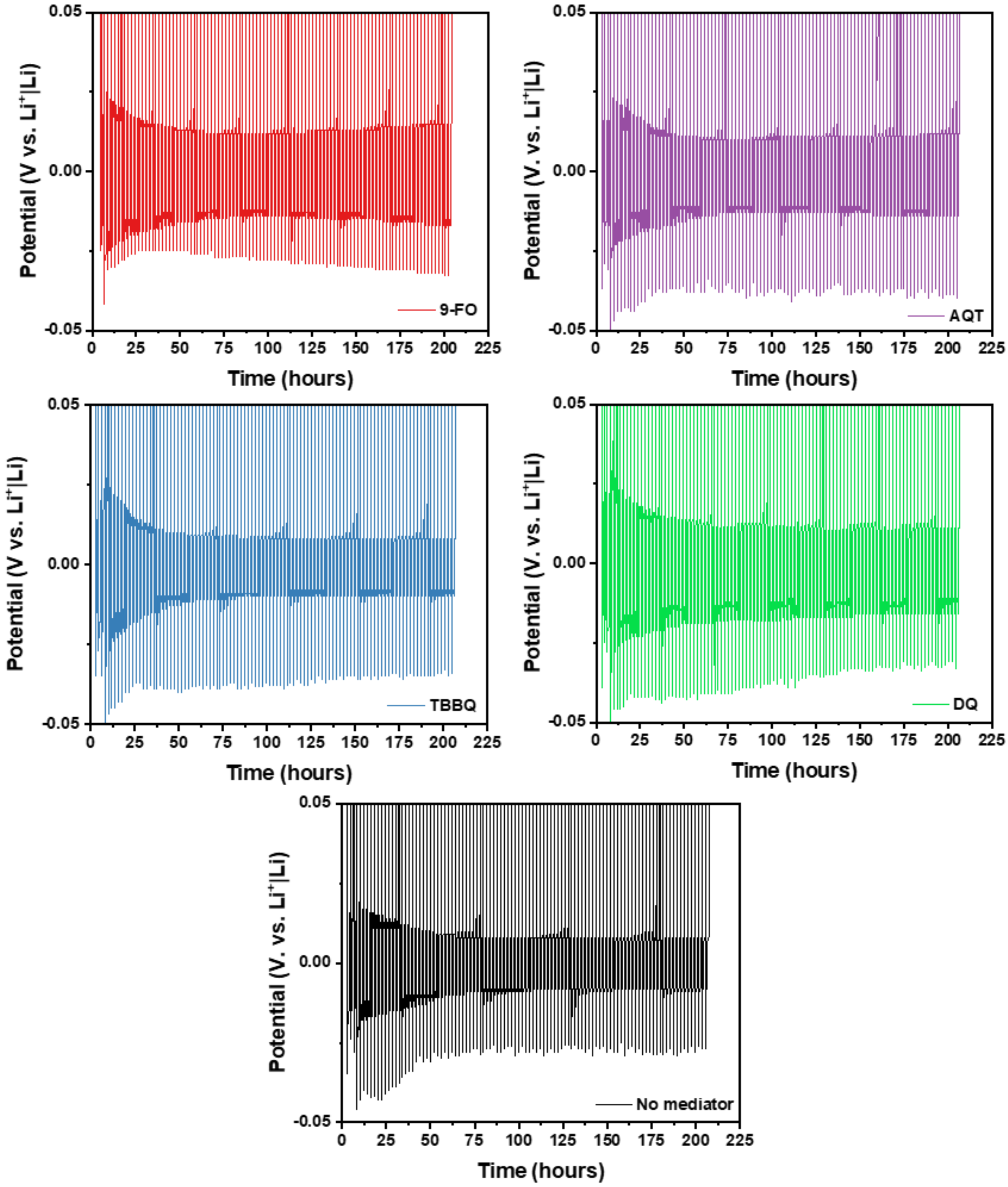

Figure S4. Potential-time plots of lithium-copper cells, with and without redox mediators. All cells had an electrolyte composition of 1.0 M LiTFSI, 0.5 M $LiNO_3$ DOL/DME (1:1 v/v). Mediated cells were doped with 50 mM redox mediator. Current densities of 0.13 mA cm^{-2} were applied for 3 cycles and 0.26 mA cm^{-2} for the remainder. Lithium plating (onto copper) was capacity limited and lithium stripping was potential limited.

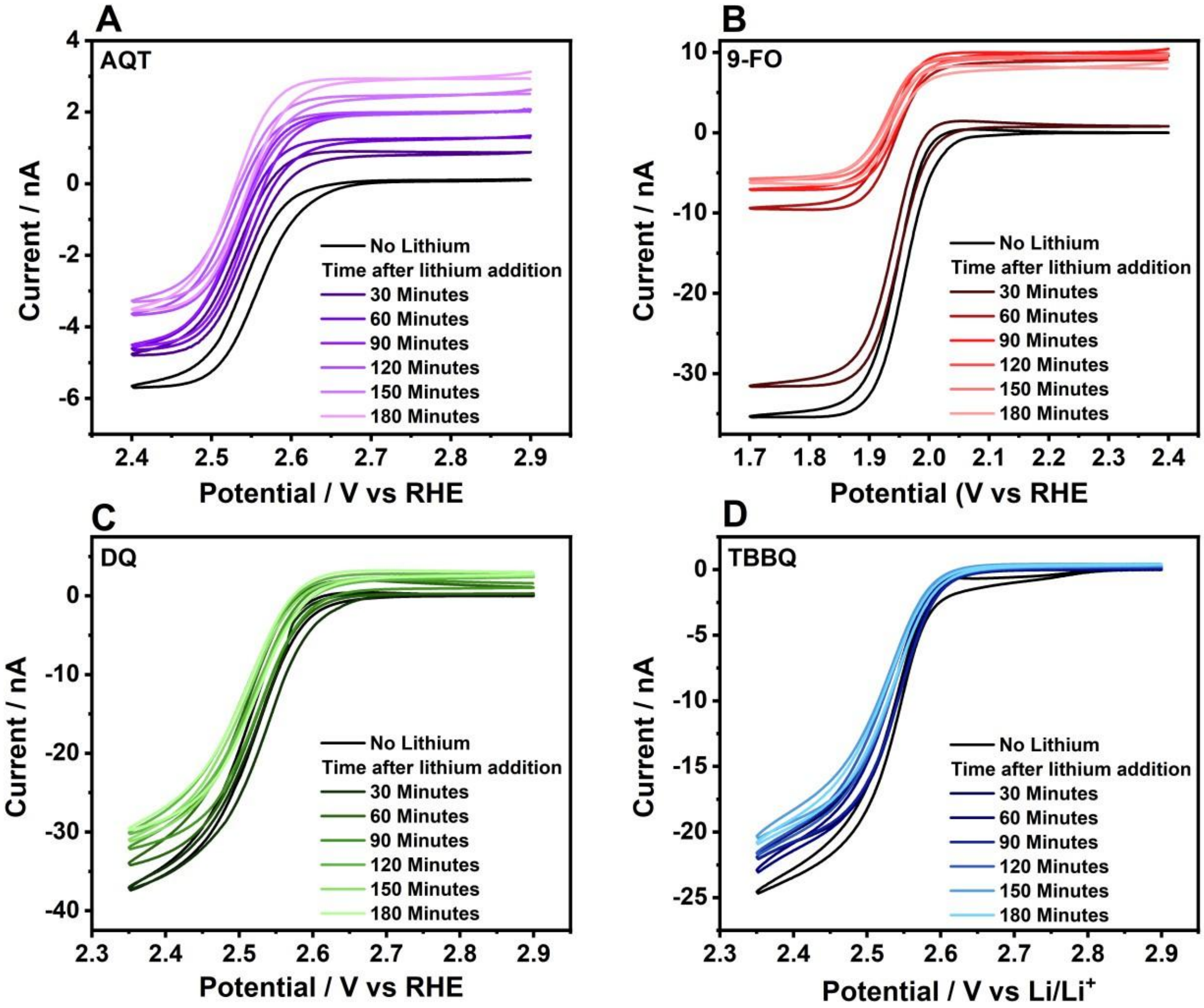

Figure S5. Ultra-micro electrode (UME) voltammograms of mediators in the presence of metallic lithium. Voltammograms of 10 mM (A) AQT, (B) 9-FO, (C) DQ, (D) TBBQ, in an electrolyte consisting of 1.0 M LiTFSI in DOL/DME (1:1 v/v), using a 25 μm diameter platinum UME. The active surface area of lithium was 3.078 cm^2 and was soaked in undoped electrolyte overnight.

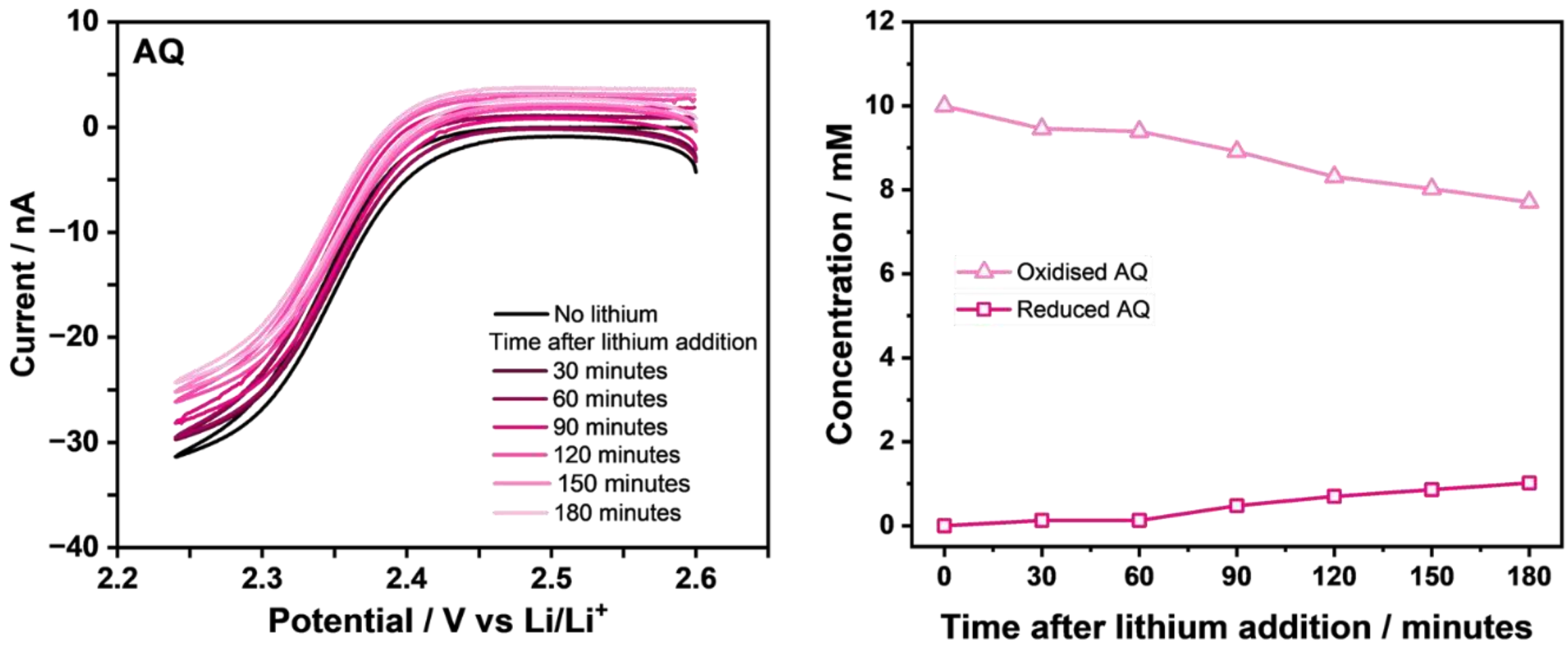

Figure S6. Anthraquinone reaction with lithium. (left) Voltammograms of 10 mM anthraquinone in an electrolyte consisting of 1.0 M LiTFSI in DOL/DME (1:1 v/v), using a 25 µm diameter platinum UME while in contact with lithium metal. The lithium metal had an active surface area of 3.078 cm^2 and was soaked in undoped electrolyte overnight to first form a stable solid electrolyte interphase; (right) Concentration vs. time plots monitoring the formation of reduced mediator after reaction of neutral mediators with Li metal over three hours, using anthraquinone.

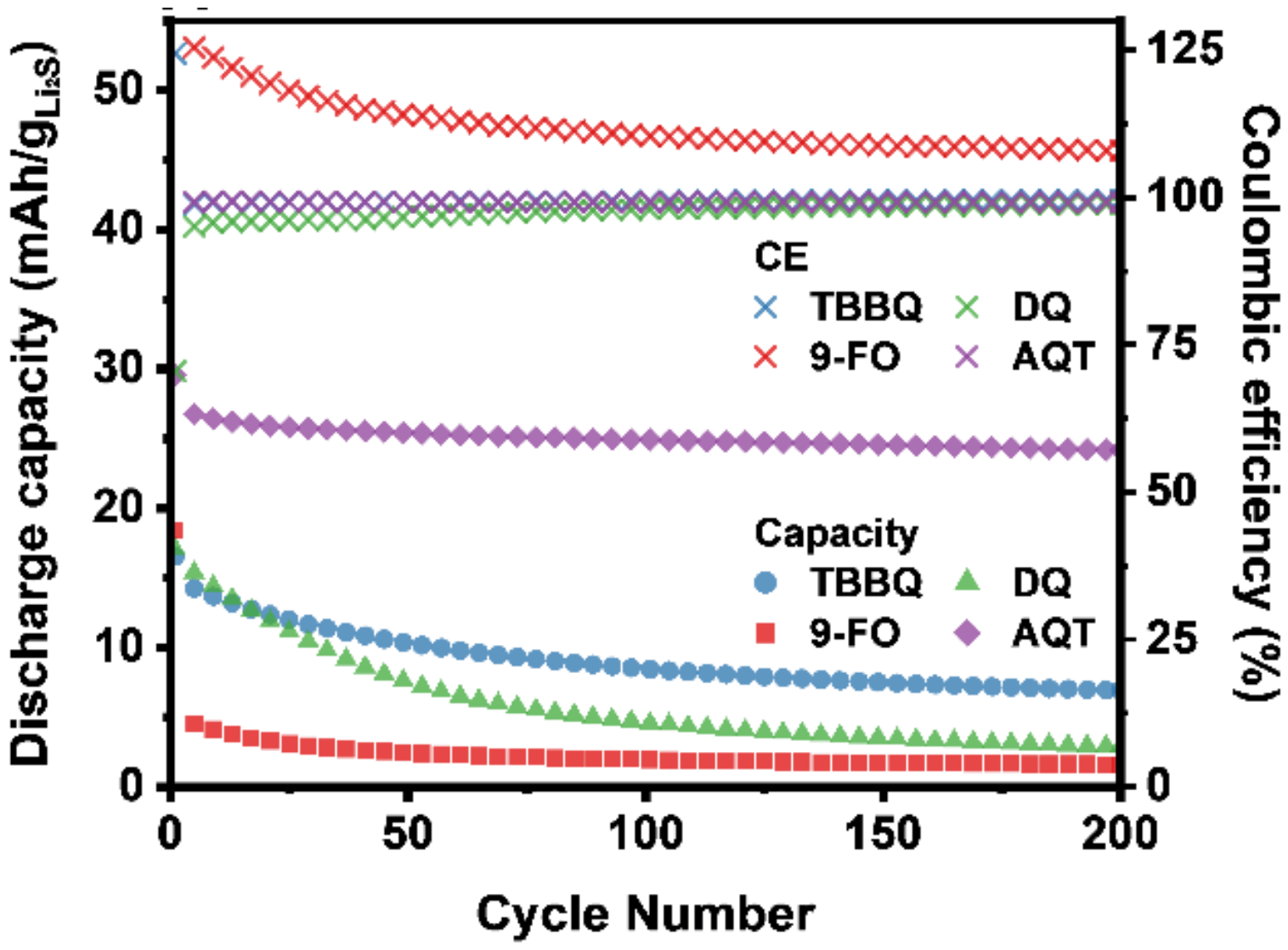

Figure S7. Mediator-doped Li-C cells showing that 9-FO and DQ are unstable. Discharge capacities (solid symbols) and coulombic efficiencies (hollow symbols) of Li-carbon cells doped with 50 mM redox mediators at a C-rate of 0.1 C. In all experiments, the electrolyte solution was 1.0 M LiTFSI, 0.5 M $LiNO_3$ DOL/DME (1:1 v/v). The C rates for all Li-carbon cells are based on the capacity obtained from a Li_2S cell with a loading of 2.5 mg.

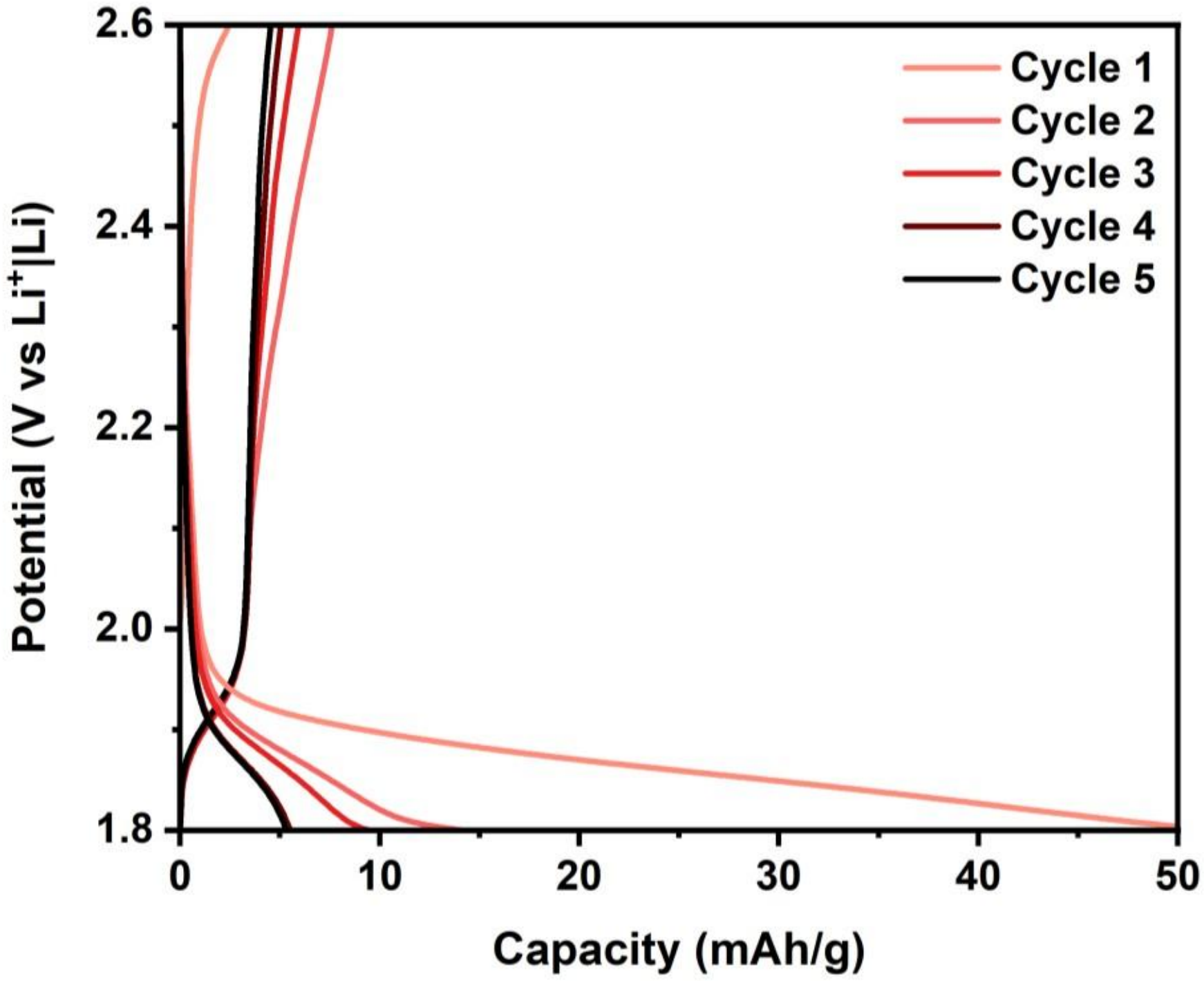

Figure S8. Li_2S cell with 50 mM 9-FO using typical Li-S voltage limits. A mass loading of 2.45 mg μ-Li_2S and electrolyte to sulfur ratios of 20 μL mg^{-1} was used. The electrolyte comprised of 1.0 M LiTFSI, 0.5 M $LiNO_3$ in DOL/DME (1:1 v/v) for the standard, with 50 mM 9-FO. The cell was cycled at 0.05 C for three cycles and 0.1 C for the remaining cycles with voltage limits between 1.8 and 2.6 V vs Li^+|Li.

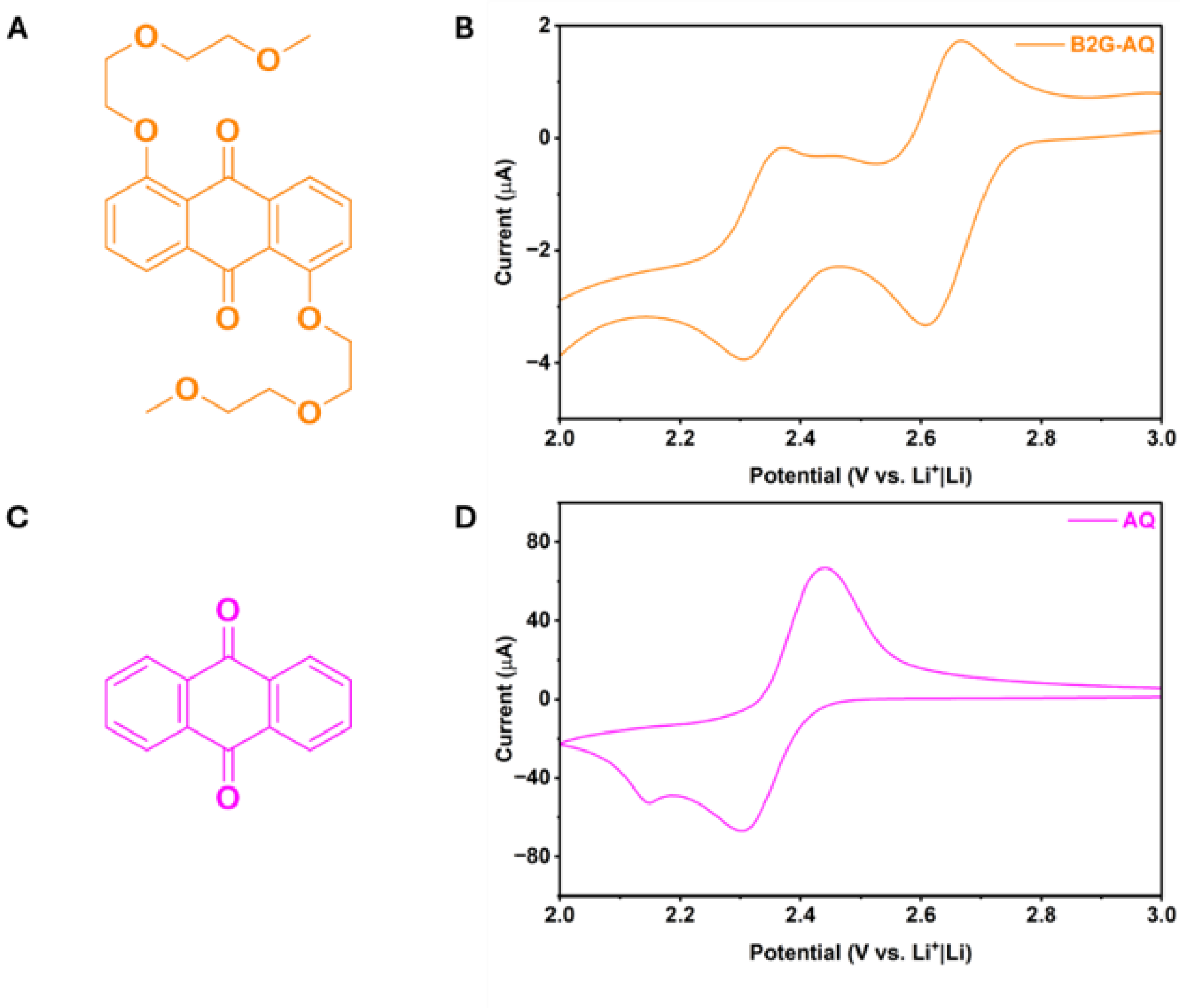

Figure S9. Structure and cyclic voltammograms of AQ and B2G-AQ. (A) Structure of B2G-AQ; (B) 1 mM B2G-AQ in 1.0 M LiTFSI in DOL/DME recorded using a 3 mm glassy carbon working electrode at a potential scan rate of 10 mV s^{-1}; (C) Structure of AQ; (D) 10 mM AQ in 1.0 M LiTFSI in DOL/DME recorded using a 3 mm gold working electrode at a potential scan rate of 100 mV s^{-1}.

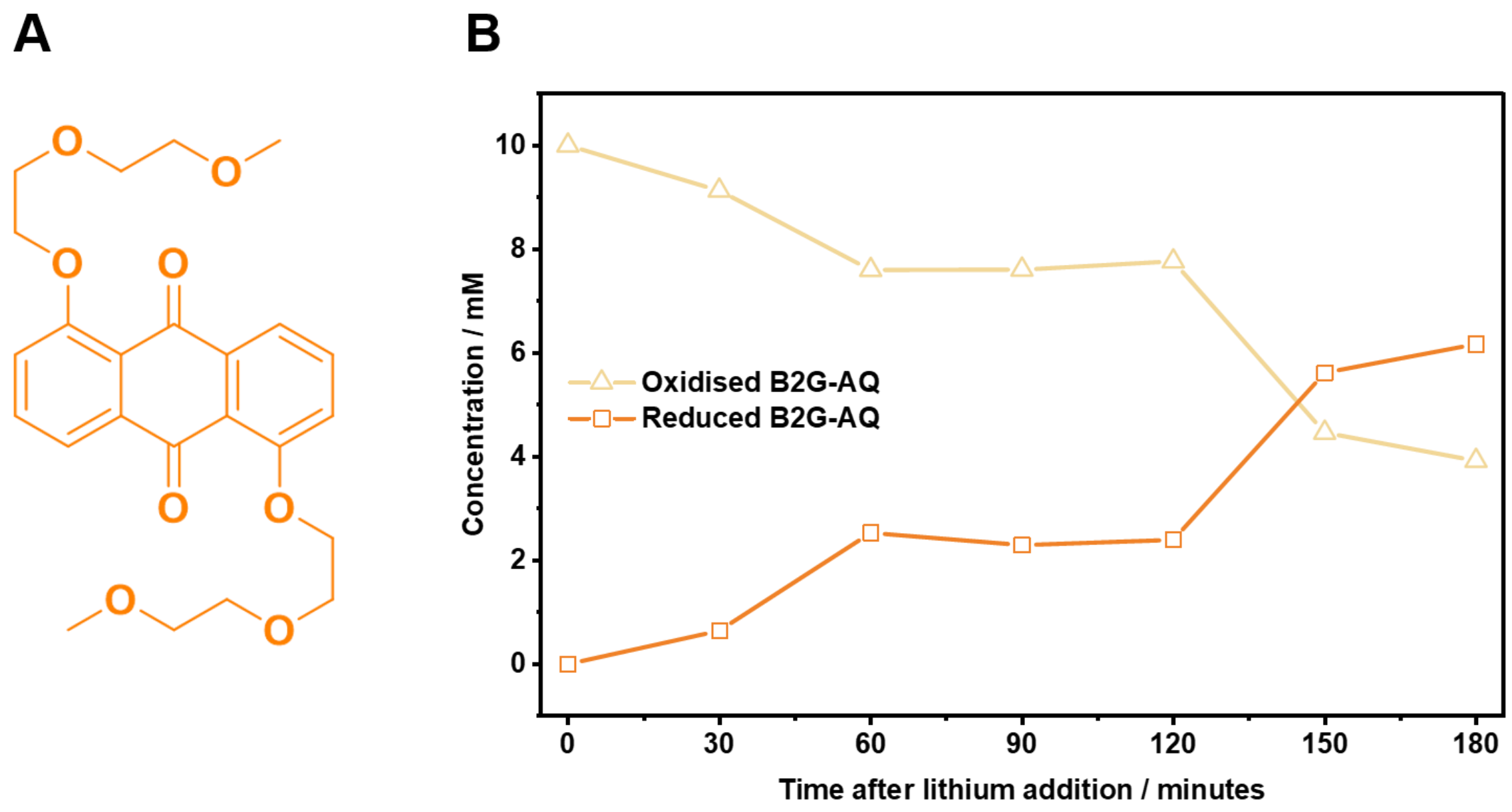

Figure S10. B2G-AQ redox mediator reaction with metallic lithium electrodes. (A) Chemical structure of B2G-AQ; (B) Concentration vs. time plot monitoring the conversion of neutral B2G-AQ to reduced B2G-AQ during reaction with Li metal, using a 10 mM mediator concentration**.** The electrolytes consisted of 1.0 M LiTFSI in DOL/DME (1:1), the active surface area of lithium was 3.078 cm^2.

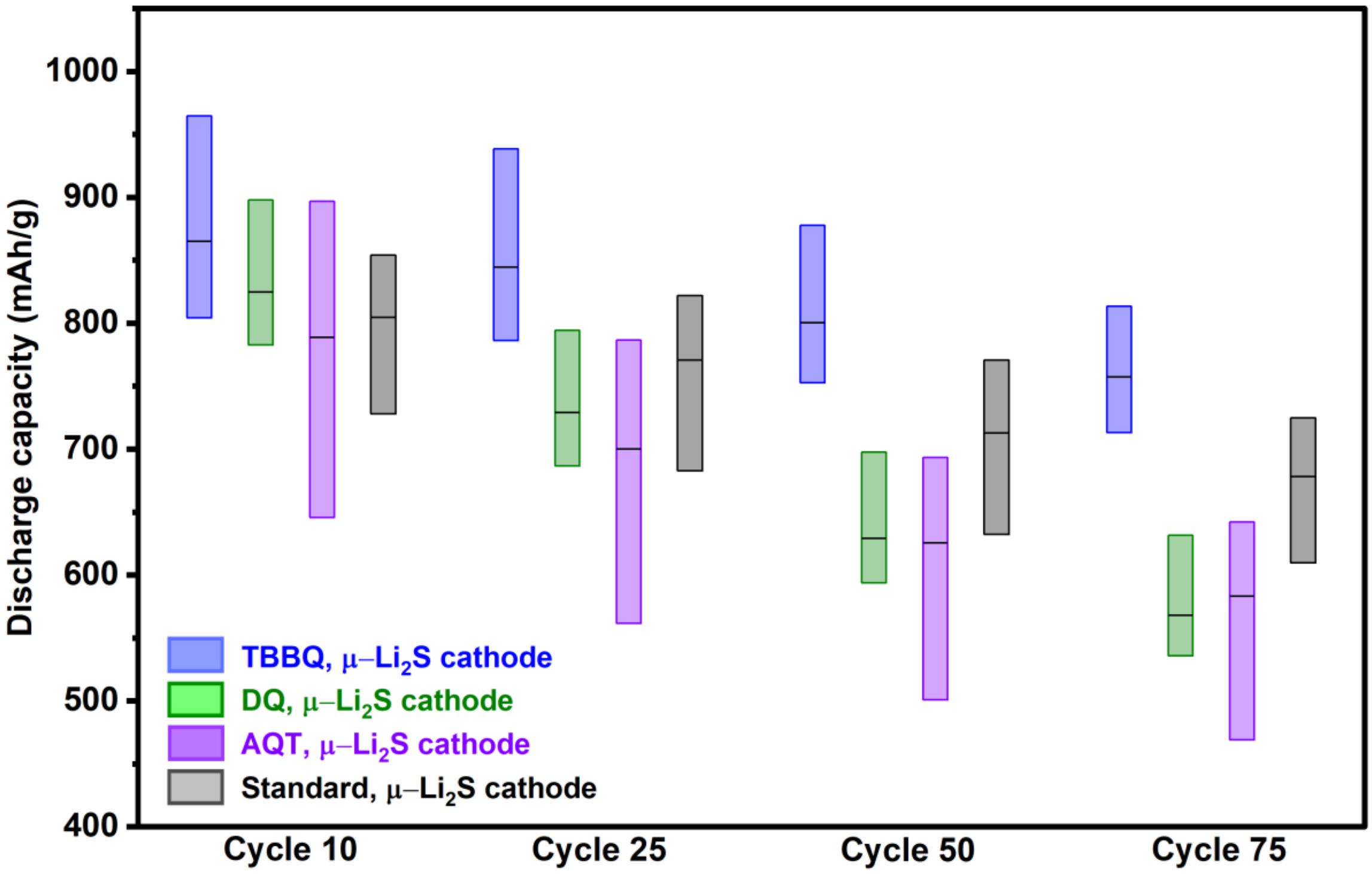

Figure S11. Cycling performance of quinone-mediated and standard cells at different cycle intervals. Box plot depicting the discharge capacities of mediated and standard cells at intervals of 10, 25, 50 and 75 cycles. The line in the middle of each box plot represents the mean value of the capacities at each cycle.

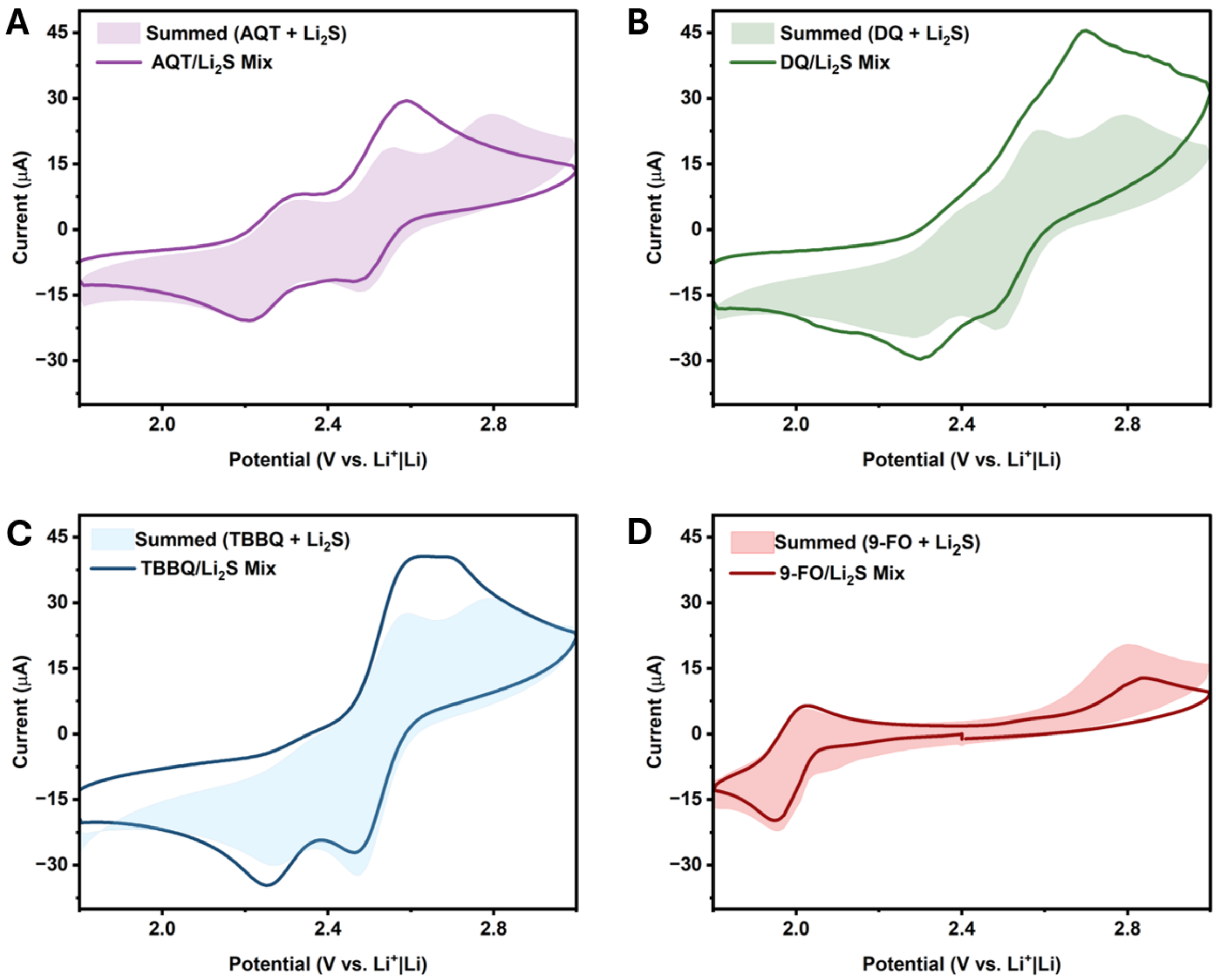

Figure S12. Cyclic voltammetry of mediators with Li_2S. Cyclic voltammograms of redox mediators mixed with Li_2S and the summed current profiles of mediators and Li_2S. The mediators used are A) AQT, B) DQ, C) TBBQ and D) 9-FO.

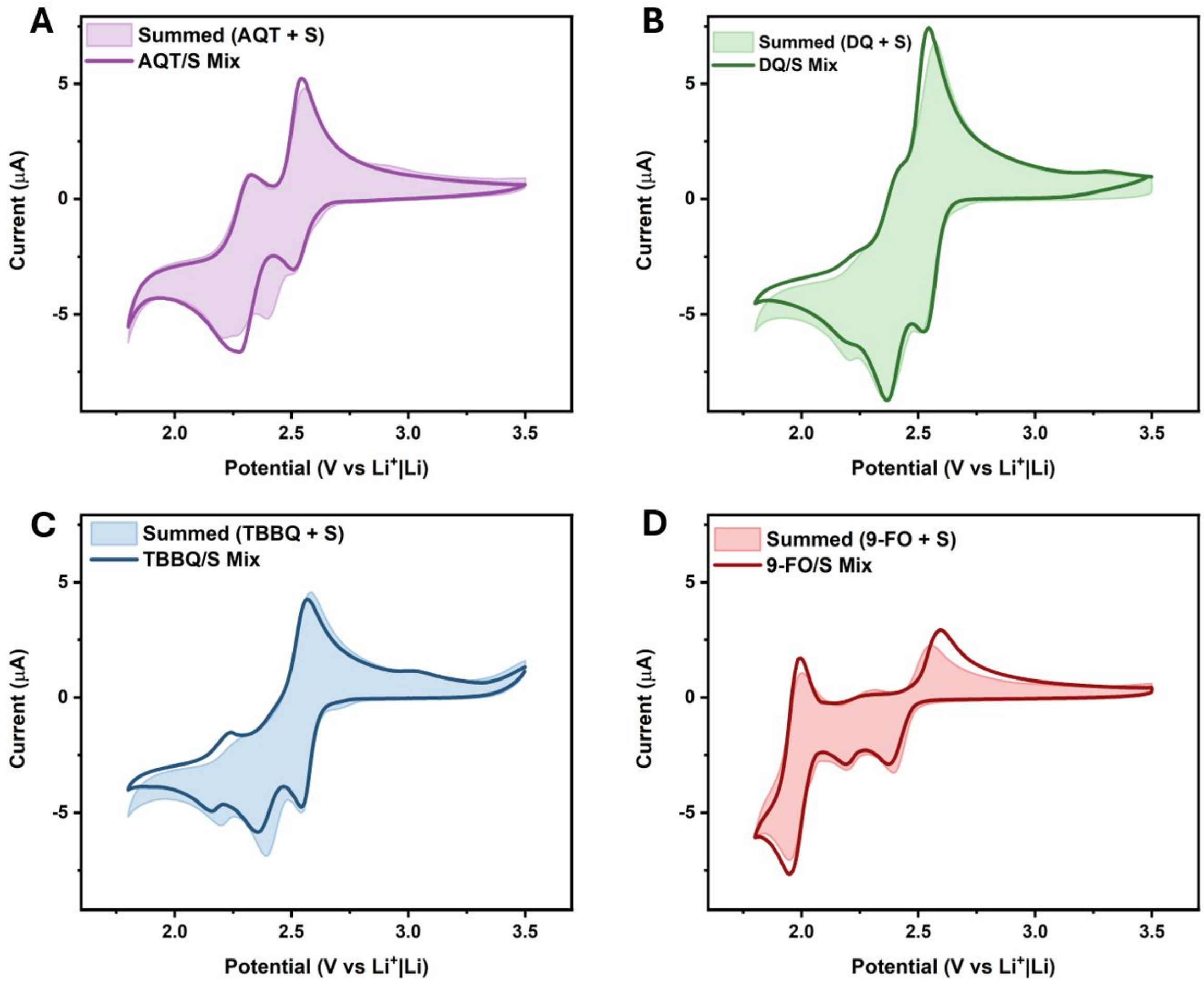

Figure S13. Cyclic voltammetry of mediators with S_8. Cyclic voltammograms of redox mediators mixed with S_8 and the summed current profiles of mediators and S_8. The mediators used are A) AQT, B) DQ, C) TBBQ and D) 9-FO.

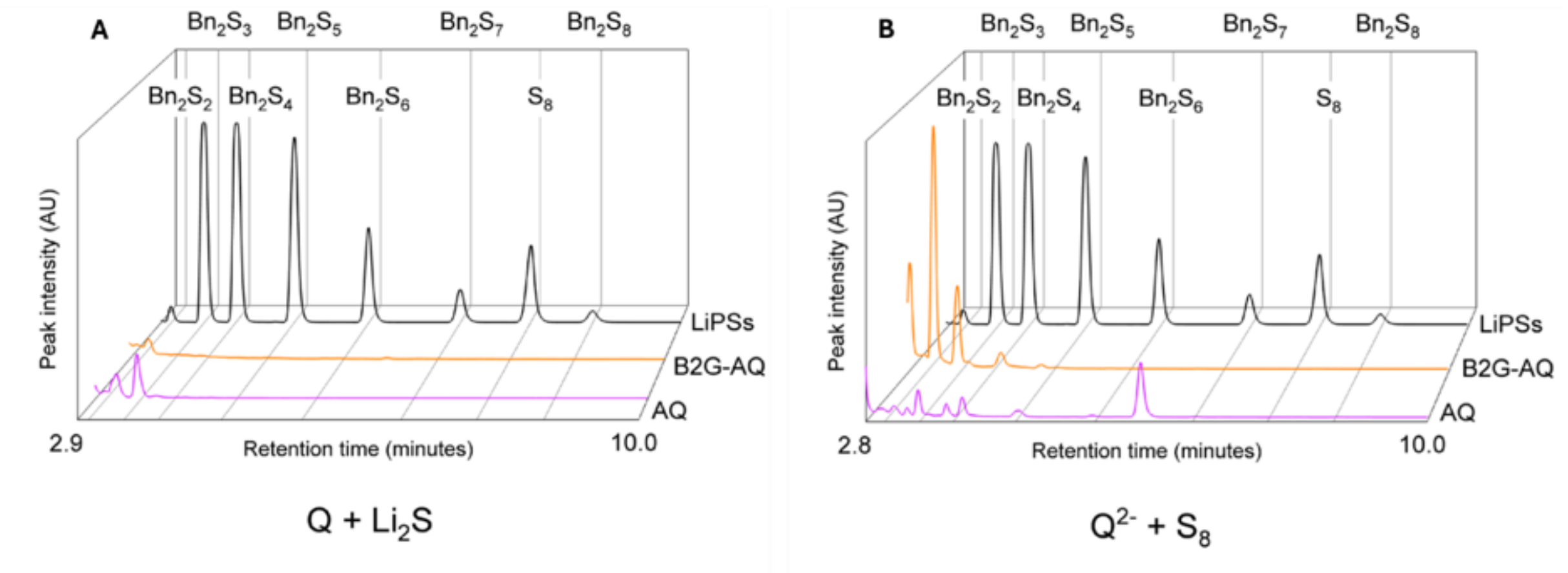

Figure S14. Liquid chromatography analysis of the reactions between redox mediators and S_8 and Li_2S using benzyl chloride as a polysulfide-capping agent. Chromatograms of (A) 10 mM redox mediators mixed with 2.3 mg (1 mM equivalent) Li_2S, and (B) 10 mM electrochemically reduced AQ and 10 mM electrochemically reduced B2G-AQ mixed with 1.28 mg (1 mM equivalent) S_8. Samples were prepared for LC-MS by combining 200 µL of the reaction solution with 200 µL BnCl and 1.0 mL methanol.

Note 1.

To further probe the relationship between quinone redox potentials and sulfur conversion, two additional quinone derivatives, b2G-AQ and anthraquinone (AQ), were also investigated (Figure SI 9). These compounds were selected to provide further variation in the positions of the first and second quinone reductions while retaining the same fundamental redox-active scaffold.

The cyclic voltammogram of b2G-AQ closely resembled that of AQT, exhibiting two well-defined reduction processes separated by approximately 300 mV. Consistent with the relatively negative potential of its second reduction, oxidation of Li_2S produced predominantly low-order polysulfides rather than elemental sulfur, whereas electrochemically generated b2G-AQ^{2-} completely consumed S_8 to form lower-order polysulfides.

In contrast, AQ displayed a distinct first reduction at approximately 2.35 V vs Li^+|Li, while the second reduction was comparatively small and exhibited characteristics consistent with surface-confined behaviour, indicative of dimerisation under the experimental conditions. Consequently, AQ effectively behaved as a one-electron mediator during the chemical reactivity studies. Consistent with its relatively negative reduction potential, AQ showed only limited oxidation of Li_2S but readily reduced S_8 to lower-order polysulfides.

Together, these additional quinones reinforce the structure-performance relationship identified above, demonstrating that while sufficiently negative reduction potentials favour sulfur reduction, efficient oxidation of Li_2S to elemental sulfur requires the second quinone reduction to occur at a sufficiently positive potential.

References

1. Tsao, Y. *et al.* Designing a Quinone-Based Redox Mediator to Facilitate Li_2S Oxidation in Li-S Batteries. *Joule* **3**, 872–884 (2019).
